# Novel Epidithiodiketopiperazine Derivatives in the Mutants of the Filamentous Fungus *Trichoderma hypoxylon*

**DOI:** 10.3390/jof11040241

**Published:** 2025-03-22

**Authors:** Zedong Ren, Yuanyuan Li, Peng-Lin Wei, Shengquan Zhang, Dong Wang, Jie Fan, Wen-Bing Yin

**Affiliations:** 1 School of Traditional Chinese Materia Medica, Shenyang Pharmaceutical University, Shenyang 110016, China; ren102323@163.com (Z.R.); dongwang@syphu.edu.cn (D.W.); 2 State Key Laboratory of Mycology, Institute of Microbiology, Chinese Academy of Sciences, Beijing 100101, China; 15008151347@163.com (Y.L.); wpl0816@126.com (P.-L.W.); 13140607588@163.com (S.Z.); 3 Medical School, University of Chinese Academy of Sciences, Beijing 100049, China; 4 Shandong Academy of Medical Sciences, Shandong First Medical University, Jinan 250117, China; 5 Laboratory of China-Korea Molecular Pharmacognosy, Shenyang Pharmaceutical University, Shenyang 110016, China

**Keywords:** epidithiodiketopiperazines, *Trichoderma hypoxylon*, gene deletion mutants, biosynthetic pathway, shunt biosynthetic pathway

## Abstract

Epidithiodiketopiperazines (ETPs) are a class of fungal secondary metabolites (SMs) featuring a transannular disulfide bridge at the diketopiperazine (DKP) core. The complex structures and biological activities have attracted widespread attention from biologists and chemists. In this study, we identified five novel ETP derivatives **4**–**7** and **4′** from three gene deletion mutants of *Trichoderma hypoxylon* CGMCC 3.17906, including Δ*tdaP*, Δ*tdaQ*, and Δ*tdaQ*Δ*tdaI*. Their structures were characterized through NMR and HR-ESI-MS data analysis. Compounds **4** and **4′** have unique heteroatom substitutions at the α and α′ positions, compound **5** possesses a unique α, β′-disulfide bridge, and compounds **6** and **7** contain a C3**′**-(thio)carbonyl group. Based on structural elucidation and biosynthetic pathway of α, β′-disulfide bridged ETPs, we also proposed the formation of **4**–**7** and **4′**. This study not only expands the chemical diversity of ETPs, but also offers new mechanistic insights into the biosynthetic pathways of fungal ETPs.

## 1. Introduction

**Figure 1:**
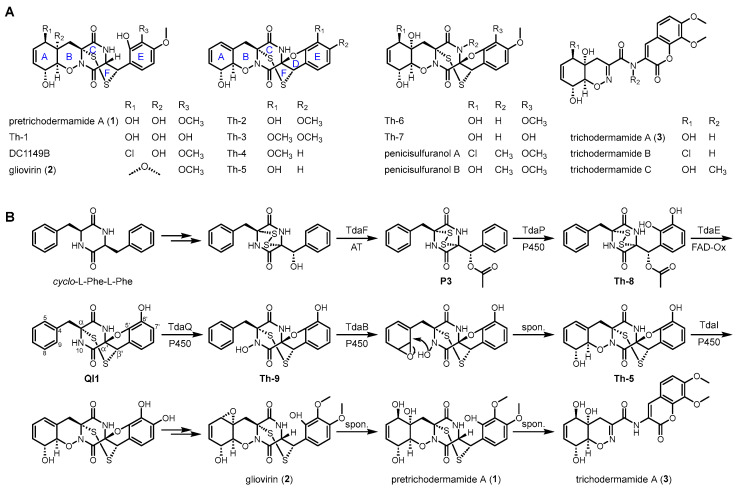
Representative epidithiodiketopiperazine (ETP) derivatives identified in fungi (**A**) and the biosynthetic pathway of pretrichodermamide A (**1**) in *Trichoderma hypoxylon* (**B**).

Epidithiodiketopiperazines (ETPs) are a unique class of fungal secondary metabolites (SMs) featuring a diketopiperazine (DKP) core and a distinctive transannular disulfide bridge [1,2,3,4,5]. These compounds are renowned for their structural diversity and potent biological activities, such as antitumor, antimicrobial, and immunosuppressive properties [6,7,8,9]. Based on the positions of sulfur atoms attached to 2,5-DKP, ETPs can be divided into two subfamilies, i.e., α, α′-disulfide (C2, C2′) and α, β′-disulfide (C2, C3′) bridged subtypes. Hundreds of α, α′-disulfide bridged ETPs have been reported in previous studies, such as gliotoxin, acetylaranotin, sirodesmin PL, chaetocin, and chetoseminudin A [10,11,12,13,14,15]. In contrast, only a few ETPs feature an irregular di(poly)sulfide that is anchored at the α and β′ positions of the DKP core, such as pretrichodermamides, aspirochlorine, lasiodipline D, and phaeosphaone D [16,17,18,19,20]. In addition to the transannular α, β′-disulfide (ring F), compounds of pretrichodermamide family share a complex heterocyclic peptide framework characterized by a 6/6/6/(5)/6/7 ring system (rings A–F) (Figure 1A) [16]. The unique structural properties confer pretrichodermamides important biological activities. Previous studies have shown that pretrichodermamides play an important role in the agriculturally beneficial fungus *Trichoderma* to inhibit the growth of plant pathogens [9,21,22,23,24]. Moreover, the majority of pretrichodermamides have been extensively studied for their potent anticancer activities [6].

The fungicolous fungus *Trichoderma hypoxylon* CGMCC 3.17906, which was isolated from the stroma of *Hypoxylon anthochroum* in Thailand, is one of the producers of pretrichodermamides [25]. In our previous studies, we have identified the prototype pretrichodermamide A (**1**), together with its precursor gliovirin (**2**) and the desulfurized derivative trichodermamide A (**3**) from *T. hypoxylon* and reported the complex biosynthesis by targeted deletions, feeding experiments, and biochemical investigations (Figure 1B and Figure S1) [16,26,27]. The formation of α, β′-disulfide requires the installation of α, α′-disulfide catalyzed by the flavin-containing enzymes TdaR, β′-acetoxy catalyzed by the acetyltransferase TdaF, and C5′, C6′-dihydroxy groups catalyzed by the cytochrome P450 monooxygenase (CYP) TdaP as prerequisites. Specifically, the flavin-containing enzyme TdaE harboring a noncanonical CXXQ motif plays a key role in the conversion of the α, α′- into the α, β′-disulfide via a reactive *ortho*-quinone methide intermediate [27]. At the late stage of α, β′-disulfide formation, the formation of **1**–**3** requires seven tailoring enzymes for the sequential 1,2-oxazine construction, phenyl modifications, cyclohexadiene oxidation, and furan opening (Figure 1B) [16].

More interestingly, the biosynthetic elucidation of **1** uncovered a highly flexible catalytic machinery of multiple enzymes, which enables the generation of even greater structural diversity than earlier studies (Figure 1A and Figure S2). Collectively, a total number of 47 structures containing 39 shunt products were identified in the single and double gene deletion mutants. However, considering the remarkable catalytic flexibility of Tda enzymes, it is plausible that more diverse products could potentially be generated in these gene deletion mutants. In this study, we optimized the cultivation conditions of three gene deletion mutants, including Δ*tdaP*, Δ*tdaQ*, and Δ*tdaQ*Δ*tdaI*, and identified five novel ETP derivatives (**4**–**7** and **4′**). We also proposed the formation of **4**–**7** and **4′** based on the biosynthetic pathway of pretrichodermamides. Our results expand the structural diversity of fungal ETPs and provide further insights into the complex biosynthetic pathways of α, β′-disulfide bridged ETPs.

## 2. Materials and Methods

### 2.1. Strain, Media, and Culture Conditions

*T*. *hypoxylon* was isolated from the stroma of *H*. *anthochroum* in Thailand and deposited in the China General Microbiological Culture Collection Center (CGMCC 3.17906, Beijing, China) [25]. The gene deletion mutants of *T. hypoxylon* used in this study, including Δ*tri5*, Δ*tdaP*Δ*tri5*, Δ*tdaQ*Δ*tri5*, Δ*tri5*Δ*thlig4*, and Δ*tdaQ*Δ*tdaI*Δ*tri5*Δ*thlig4* mutants, designated as TYHL26, TYHL41, TYPL43, TYYH3, and TYPL52, respectively, are summarized in Table S1. All strains were cultivated at 26 °C on potato dextrose agar (PDA, BD Difco™, 39 g/L) for growth, and on rice medium for detection and isolation of differential SMs.

### 2.2. Large-Scale Fermentation, Extraction, and Isolation of Differential Compounds

To isolate **4** and **4′**, Δ*tdaP*Δ*tri5* mutant TYHL41 was cultivated in a 200 × 500-mL flask containing 100 g rice and 150 mL H_2_O at 26 °C for 11 days. The rice cultures were extracted with 4 L ethyl acetate (EtOAc) three times and concentrated under reduced pressure to give a crude extract (29.3 g), which was dissolved in 300 mL acetonitrile (ACN)/H_2_O (9:1, *v*/*v*) and extracted with an equal volume of *n*-hexane to remove oily constituents to afford the sub-crude extract (16.5 g). The sub-crude extract was subjected to silica gel column chromatography (CC) (200–300 mesh) and eluted with a stepwise gradient of petroleum ether (PE)/EtOAc (1:0, 6:1, 5:1, 4:1, 2:1, 1:1, 1:2, and 0:1, *v*/*v*) to yield 44 fractions (1–44). Fractions 37–41 were then eluted with 1.8 L PE/EtOAc (1:2, *v*/*v*). Further separation and purification of fraction 38 was carried out by semipreparative HPLC with ACN/H_2_O (45:55, *v*/*v*) at a flow rate of 2.5 mL/min to yield the mixture of **4** and **4′** (2.0 mg).

To isolate **5**, Δ*tdaQ*Δ*tri5* mutant TYPL43 was cultivated in a 200 × 500-mL flask containing 50 g rice and 75 mL H_2_O at 26 °C for 11 days. The rice cultures were extracted with 4 L EtOAc three times and concentrated under reduced pressure to give a crude extract (30.0 g), which was dissolved in 400 mL ACN/H_2_O (9:1, *v*/*v*) and extracted with an equal volume of *n*-hexane to remove oily constituents to afford the sub-crude extract (17.2 g). The sub-crude extract was subjected to silica gel column chromatography (CC) (200–300 mesh) and eluted with a stepwise gradient of PE/EtOAc (1:0, 3:1, 2:1, 1:1, 1:2, 1:3, and 0:1, *v*/*v*) to yield 55 fractions (1–55). Fractions 20–36 were then eluted with 3.5 L PE/EtOAc (1:1, *v*/*v*). Further separation and purification of fraction 31 was carried out by semipreparative HPLC with ACN/H_2_O (25:75, *v*/*v*) at a flow rate of 2.5 mL/min to yield **5** (1.7 mg).

To isolate **6** and **7**, Δ*tdaQ*Δ*tdaI*Δ*tri5*Δ*thlig4* mutant TYPL52 was cultivated in a 200 × 500-mL flask containing 50 g rice and 75 mL H_2_O at 26 °C for 9 days. The rice cultures were extracted with 4 L EtOAc three times and concentrated under reduced pressure to give a crude extract (25.5 g), which was dissolved in 200 mL ACN/H_2_O (9:1, *v*/*v*) and extracted with an equal volume of *n*-hexane to remove oily constituents to afford the sub-crude extract (13.0 g). The sub-crude extract was subjected to silica gel column chromatography (CC) (200–300 mesh) and eluted with a stepwise gradient of PE/EtOAc (1:0, 5:1, 3:1, 2:1, 1:1, 1:2, and 0:1, *v*/*v*) to yield 32 fractions (1–32). Fractions 20–25 were then eluted with 2 L PE/EtOAc (1:1, *v*/*v*). Further separation and purification of fraction 21 was carried out by semipreparative HPLC with ACN/H_2_O (40:60, *v*/*v*) at a flow rate of 2.5 mL/min to yield **6** (1.0 mg). Similarly, fraction 22 was processed through semipreparative HPLC under identical conditions, resulting in the isolation of compound **7** (1.5 mg).

### 2.3. HPLC and LC-MS Analysis

Semipreparative purification on HPLC was performed on an SSI HPLC system (Teledyne SSI Lab Alliance Series III pump system and Series 1500 Photodiode Array Detector, Torrance, CA, USA) with an Eclipse XDB-C18 column (250 × 9.4 mm, 5 μm, Aligent, Santa Clara, CA, USA) at a flow rate of 2.5 mL/min. UV absorptions at 230 nm, 254 nm, and 340 nm are illustrated.

LC-MS analysis was performed on an Agilent HPLC 1200 series system equipped with a single quadrupole mass selective detector and an Agilent 1100LC MSD model G1946D mass spectrometer by using a Venusil XBP C18 column (3.0 by 50 mm, 3 μm, Bonna-Agela Technologies, Tianjin, China). Water (A) with 0.1% (*v*/*v*) formic acid and ACN (B) were used as the solvents at a flow rate of 0.5 mL/min. The substances were eluted with a linear gradient from 5 to 100% B in 30 min, then washed with 100% (*v*/*v*) solvent B for 5 min, and equilibrated with 5% (*v*/*v*) solvent B for 10 min. The mass spectrometer was set in electrospray positive ion mode for ionization. HR-ESI-MS data were recorded on an Agilent Technologies 6520 Accurate-Mass Q-TOF LC/MS spectrometer equipped with an electrospray ionization (ESI) source. The HR-ESI-MS data of compounds analyzed in this study are available at the GNPS website (https://massive.ucsd.edu, accessed on 7 March 2025) under MassIVE ID number MSV000091394.

### 2.4. NMR Analysis

NMR spectra were recorded on a Bruker Avance-500 MHz spectrometer at room temperature (Bruker Corporation, Karlsruhe, Germany). All spectra were processed with MestReNova 12.0.3 (Metrelab). Chemical shifts are referenced to those of the solvent signals.

## 3. Results

### 3.1. Analysis of Differential Compounds in the Gene Deletion Mutants of T. hypoxylon

**Figure 2:**
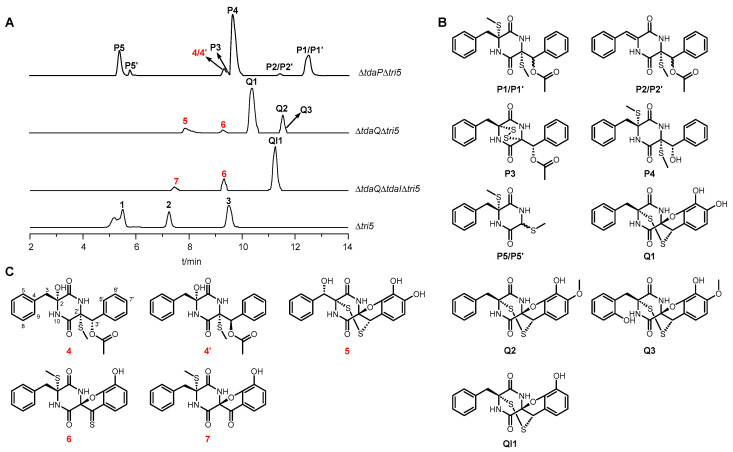
Differential compounds identified from single gene deletion mutants of *tdaP* and *tdaQ* and double gene deletion mutants of *tdaQ* and *tdaI* in the background of *Trichoderma hypoxylon* Δ*tri5*, which was previously constructed for the elimination of the predominant secondary metabolites (SMs), trichothecenes of wild type (WT) [28]. (**A**) LC-MS analysis of the crude extracts of Δ*tdaP*Δ*tri5*, Δ*tdaQ*Δ*tri5*, Δ*tdaQ*Δ*tdaI*Δ*tri5*, and Δ*tri5* mutants. (**B**) Twelve ETP derivatives identified from the three mutants in our previous studies [16,27]. (**C**) Five novel ETP derivatives, including **4**–**7** and **4′**, identified from the three mutants in this study. **1**–**3** are the final ETP products of *tda* gene cluster. EICs refer to [M+H]^+^ ions of **1**–**3**, together with [M+Na]^+^ ions of **4**–**7**, **4′**, **P1**–P**5**, **P1′**, **P2′**, **P5′**, **Q1**–**Q3**, and **QI1** with tolerance ranges of ±0.25. The new compounds identified in this study are indicated in red.

In previous studies, we have constructed single and double gene deletion mutants of *tda* genes to identify the biosynthetic intermediates and elucidate the biosynthesis of α, β′-disulfide bridged ETPs (Figure 1B) [16,26,27]. Single deletions of four *tda* genes, which encode for CYP TdaP, acyltransferase TdaF, and flavin-containing enzymes TdaR and TdaE, respectively, led to the identification of 20 novel ETP derivatives formed before the formation of the α, β′-disulfide [27]. Deletion of the CYP-encoding gene *tdaP* completely abolished the formation of **1**–**3** and led to the accumulation of **P1**–**P5**, **P1′**, **P2′**, and **P5′** (Figure 2). **P1** and **P1′** are a pair of isomers characterized by thiomethyl groups at both α and α′ positions, as well as a β′-acetoxy group. Upon demethylthiolation, **P1** and **P1′** are transformed into another pair of isomers, namely **P2** and **P2′**. **P3** was deduced as the precursor of TdaP in the biosynthetic pathway of **1**–**3** carrying α, α′-disulfide and β′-acetoxy group. **P4** was identified in Δ*tdaR* mutant and can be converted to **P5** and **P5′** via the spontaneous retro-aldol reaction. At the late stage of α, β′-disulfide formation, two CYPs TdaQ and TdaB collaboratively catalyze the construction of 1,2-oxazine. Deletion of *tdaQ* eliminated the hydroxylation at N10, thereby disrupting the construction of 1, 2-oxazine [16]. Three shunt products **Q1**–**Q3** were isolated from Δ*tdaQ* mutant and identified as α, β′-disulfide-containing derivatives. Among these compounds, **Q2** is distinguished from **Q1** by the presence of a methoxy group at the C7′ position, while **Q3** differs from **Q2** through hydroxylation at the C9 position. Furthermore, double gene deletion of *tdaQ* and *tdaI* led to the accumulation and subsequent identification of **QI1**, which serves as the biosynthetic precursor for **Q1**–**Q3** (Figure 2).

To identify more differential SMs in *tda* gene deletion mutants, we optimized the cultivation conditions of Δ*tdaP*, Δ*tdaQ*, and Δ*tdaQ*Δ*tdaI* in this study. These three strains were cultivated on rice media at 26 °C for 11, 11, and 9 days, respectively. Subsequently, we extracted the cultures with EtOAc and concentrated them under reduced pressure to give the crude extracts. LC-MS analysis of the culture supernatants revealed not only the previously identified compounds but also several novel differential product peaks (Figure 2A). With the exception of **P1**–**P5** and **P1′**–**P3′**, cultivation of Δ*tdaP* mutant resulted in the accumulation of a novel product peak **4** eluting at 9.1 min with an [M+Na]^+^ ion at *m*/*z* 437.1 ± 0.25. Cultivation of Δ*tdaQ* mutant resulted in the accumulation of the previously identified **Q1**–**Q3** and two novel product peaks, i.e., **5** eluting at 7.9 min with an [M+Na]^+^ ion at *m*/*z* 441.0 ± 0.25 and **6** eluting at 9.2 min with an [M+Na]^+^ ion at *m*/*z* 423.0 ± 0.25. Double gene deletion mutant of *tdaQ* and *tdaI* also resulted in the accumulation of the product peak of **6**, together with another product peak **7** eluting at 7.5 min with an [M+Na]^+^ ion at *m*/*z* 407.1 ± 0.25. Then, we performed targeted isolation using a combination of normal-phase silica gel chromatography, and semi-preparative HPLC, leading to the identification of five novel ETP derivatives, i.e., **4**–**7** and **4′**.

### 3.2. Characterization of Five Novel ETP Derivatives from Gene Deletion Mutants of T. hypoxylon

**Figure 3:**
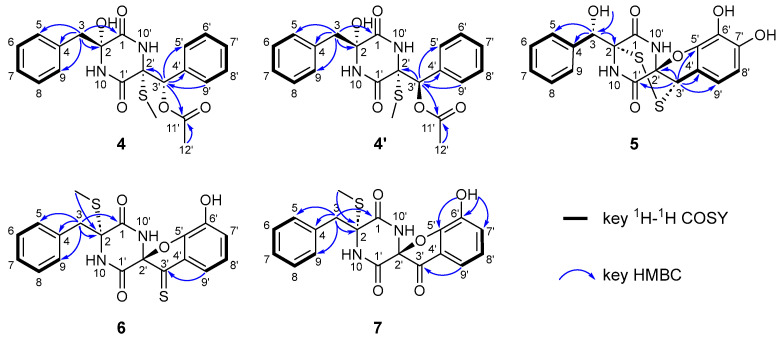
Key ^1^H-^1^H COSY and HMBC correlations of compounds **4**–**7** and **4′**.

The white powder compound was isolated from the gene deletion mutant of *tdaP*, and its molecular formula, C_21_H_22_N_2_O_5_S, was deduced from HR-ESI-MS analysis showing an [M+Na]^+^ ion at *m*/*z* 437.1149 (calcd. 437.1142), indicating twelve unsaturation degrees (Table S2). The ^1^H and ^13^C NMR spectroscopic analysis revealed that the sample contained a mixture of compounds, suggesting the presence of a pair of isomers (**4** and **4′**) (Table 1 and Figure S3). Three carbonyl carbons (δ_C_ 167.1, 165.3, and 168.7), two imines (δ_H_ 9.14 and 7.81), and two phenyl moieties were detected in compound **4**, suggesting the structural framework as a cyclo-l-Phe-l-Phe (cFF). The attachment of a benzyl moiety to C2 was determined by the HMBC correlations from H_2_-3 to C1, C2, C4, C5, and C9, and ^1^H-^1^H COSY correlations of H-6 with H-5 and H-7, and H-8 with H-7 and H-9, as well as the chemical shifts of C4 (δ_C_ 135.4), C5 (δ_C_ 130.8), C6 (δ_C_ 128.3), C7 (δ_C_ 128.7), C8 (δ_C_ 128.3), and C9 (δ_C_ 130.8) (Figure 3). The attachment of phenyl moiety to C3′ was determined by the HMBC correlations from H-3′ to C4′, C5′, and C9′, and ^1^H-^1^H COSY correlations of H-6′ with H-5′ and H-7′, and H-8′ with H-7′ and H-9′, as well as the chemical shifts of C4′ (δ_C_ 134.1), C5′ (δ_C_ 128.0), C6′ (δ_C_ 127.9), C7′ (δ_C_ 126.7), C8′ (δ_C_ 127.9), and C-9′ (δ_C_ 128.0). Furthermore, the HMBC correlations from H-3′ and H_3_-12′ to C11′, respectively, together with the downfield chemical shift of C3′ (δ_C_ 77.2) assigned the carbonyl group at C11 and indicated the connection between C3′ and C12 via an ester bond. The thiomethyl group attached to C2′ was confirmed by the HMBC correlations from H_3_-SCH_3_ (δ_H_ 0.86) to C2′ (δ_C_ 71.2). These results indicated that compound **4** carries one thiomethyl group at C2′ and an acetoxy group at C3′, which are identical with those in the previously identified compounds **P1** and **P1′** [27]. However, compound **4** differs from **P1**/**P1′** by the presence of a hydroxy group (δ_H_ 6.74) at C2 (δ_C_ 82.6). As a result, comprehensive NMR analysis indicated compound **4** as a novel derivative from cFF, which carries a hydroxy group at C2, a thiomethyl group at C2′, and an acetoxy group at C3′. Like **P1** and **P1′**, compounds **4** and **4′** are a pair of isomers bearing different configurations of β′-acetoxy groups.

Compound **5**, obtained as a white powder, was isolated from a gene deletion mutant of *tdaQ* and assigned the molecular formula C_18_H_14_N_2_O_6_S_2_ based on HR-ESI-MS analysis showing an [M+Na]^+^ ion at *m*/*z* 441.0188 (calcd. 441.0185), corresponding to 13 unsaturation degrees (Table S2). Comprehensive NMR analysis revealed that compound **5** was an ETP derivative from cFF, displaying characteristic DKP signals such as two carbonyl carbons (δ_C_ 167.3 and 165.4) and two imine protons (δ_H_ 9.48 and 9.34) (Table 1 and Figure S4). Similar to the previously identified **Q1**–**Q3**, compound **5** bears transannular disulfide bridge attached to C2 (δ_C_ 74.6) and C3′ (δ_C_ 54.4) positions, together with the spirofuran, which can be determined by the carbon signals of C2′ (δ_C_ 96.3) and C5′ (δ_C_ 146.3) [16]. However, only one singlet proton signal (δ_H_ 5.11) was observed being attached to the C3 position (δ_C_ 71.6) and showed clear correlations to C2 (δ_C_ 74.6), C4 (139.6), and C9 (δ_C_ 128.9) in 2D NMR spectra (Figure 3). The hydroxy group at C3 was then confirmed by the HMBC correlation from δ_H_ 6.19 to δ_C_ 71.6. In addition, comparative analysis with the NMR data of **Q1** suggested the presence of two additional hydroxy groups at C6′ (δ_C_ 129.7) and C7′ (δ_C_ 148.3). Based on these findings, compound **5** was identified as a novel ETP derivative featuring an α, β′-disulfide bridge, a spirofuran moiety, and dihydroxy substitutions at C3 and C7′.

Compounds **6** and **7** were isolated from the double gene deletion mutant of *tdaQ* and *tdaI*. Compound **6**, obtained as a yellow powder, was assigned the molecular formula C_19_H_16_N_2_O_4_S_2_ based on HR-ESI-MS analysis showing an [M+Na]^+^ ion at *m*/*z* 423.0453 (calcd. 423.0444), corresponding to 13 unsaturation degrees (Table S2). Similar to **4**, **4′**, and **5**, compound **6** is also derived from the cFF skeleton showing the characteristic DKP signals such as two carbonyl carbons (δ_C_ 165.8 and 160.2) and two imine protons (δ_H_ 9.54 and 9.49) (Table 2 and Figure S5). Comparative NMR analysis between compound **6** and the previously identified **QI1** revealed distinct structural features: (1) the absence of an α, β′-disulfide bridge, (2) the presence of a spirofuran ring, and (3) a thiomethyl group at C2, as evidenced by HMBC correlations from δ_H_ 2.3 to δ_C_ 67.6 and ^1^H-^1^H COSY correlations of δ_H_ 2.3 with δ_H_ 13.5 (Figure 3) [27]. The C3-methylene protons (δ_H_ 3.04 and 3.50) showed HMBC correlations to C1 (δ_C_ 165.8), C2 (δ_C_ 67.6), C4 (δ_C_ 134.3), C5 (δ_C_ 130.4), and C9 (δ_C_ 130.4). Interestingly, the ^13^C NMR spectrum of compound **6** displayed a characteristic thiocarbonyl signal at δ_C_ 227.5. Careful analysis of the HMBC spectrum allowed us to assign the C3′-thiocarbonyl group through a clear correlation from H-9′ (δ_H_ 7.08) of the phenyl moiety to C3′ (δ_C_ 227.5). Based on these findings, compound **6** was identified as a novel cFF derivative featuring a C2-thiomethyl group, a C3′-thiocarbonyl group, and a spirofuran ring system.

Compound **7**, obtained as a yellow powder, was assigned the molecular formula C_19_H_16_N_2_O_5_S based on HR-ESI-MS analysis showing an [M+Na]^+^ ion at *m*/*z* 407.0674 (calcd. 407.0672), corresponding to 13 unsaturation degrees (Table S2). The ^1^H and ^13^C NMR data of **7** were very similar to those of **6**, with the exception of the presence of a carbonyl group at C3′ (δ_C_ 194.9) instead of the thiocarbonyl group in **6** (δ_C_ 227.5) (Table 2 and Figure S6). HMBC analysis showed a clear correlation from H-9′ (δ_H_ 7.03) of the phenyl moiety to C3′-carbonyl group (δ_C_ 194.9) (Figure 3). Thus, compound **7** was identified as a novel cFF derivative featuring a C2-thiomethyl group, a C3′-carbonyl group, and a spirofuran ring system.

### 3.3. Plausible Biosynthetic Pathway of Compounds ***4**–**7*** and ***4′***

**Scheme 1:**
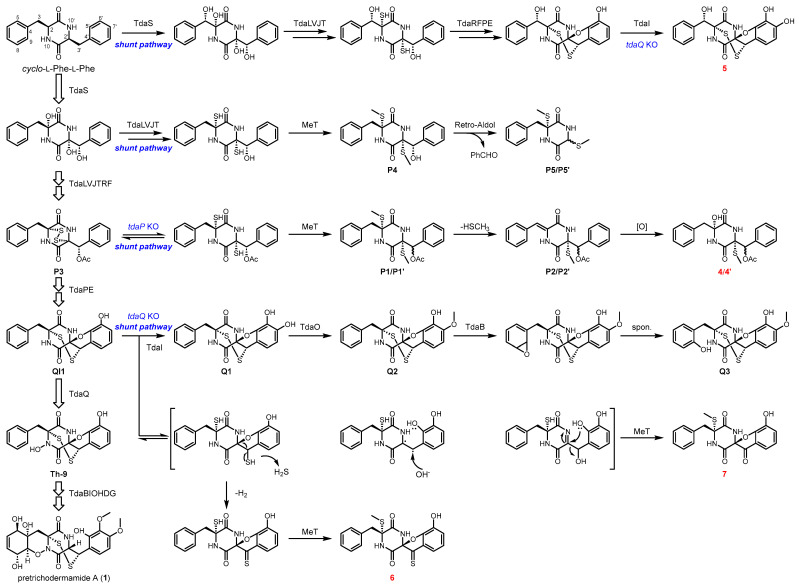
Plausible formation of compounds **4**–**7** and **4′** in *Trichoderma hypoxylon*. The new compounds identified in this study are indicated in red.

As previously reported, we have elucidated the biosynthetic pathway of **1**–**3** for understanding the formation of α, β′-disulfide bridged ETPs [16,27]. The nonribosomal peptide synthetase (NRPS) TdaA catalyzes the coupling of two units of l-Phe to afford cFF, which is further oxidized at α, α′, and β′ positions by the CYP TdaS (Scheme 1). In analogy to the biosynthesis of gliotoxin, a four-enzyme cascade by TdaL, TdaV, TdaJ, and TdaT is responsible for GSH addition and the subsequent degradation [2,29,30,31,32,33]. Then, TdaR, TdaF, and TdaP catalyze the installation of α, α′-disulfide, β′-acetoxy, and C5′, C6′-dihydroxy groups, respectively. TdaE harboring the noncanonical CMYQ motif further catalyzes the migration of α, α′-disulfide to give **QI1** carrying both α, β′-disulfide and spirofuran. At the late stage of α, β′-disulfide formation, two CYPs TdaB and TdaQ are responsible for C8, C9-epoxidation and N-hydroxylation, respectively, facilitating spontaneous cyclization to yield 1,2-oxazine. Subsequently, the CYP TdaI and methyltransferases TdaO and TdaH catalyze the formation of C6′, C7′-methoxy groups at the phenyl moiety. Further oxidation by the CYP TdaG and reduction by TdaD complete the biosynthesis of **1**.

Considering the characterized functions of Tda enzymes, we proposed the formation of **4**–**7** and **4′** in this study (Scheme 1). Compound **P3** that accumulates in the Δ*tdaP* mutant can be converted into the α, α‘-dithiol adduct through reduction. This adduct would be methylated to produce **P1**/**P1′**, which is further reduced to yield **P2**/**P2′**. Then, another oxygenase catalyzes the hydroxylation at the C2 position to afford **4**/**4′**. Compound **5**, which was identified in Δ*tdaQ* mutant, differs from **Q1** at the C3-hydroxy group. We speculate that the CYP TdaS would also catalyze the high oxidation of cFF at α, α′, β, and β′ positions, which is similar to the catalysis of AclC in aspirochlorine biosynthesis [5,34,35]. In the single gene deletion mutant of *tdaQ* and double gene deletion mutant of *tdaQ* and *tdaI*, **QI1** can be spontaneously transformed into the α, α′-dithiol intermediate, facilitating the electron transfer and hydrogen sulfide elimination, ultimately leading to the generation of a carbonyl group in compound **7**. The α, α′-dithiol intermediate can also be alternatively reduced to give the thiocarbonyl group in compound **6**.

## 4. Conclusions

Taken together, through comprehensive analysis and systematic isolation of three *tda* gene deletion mutants, including Δ*tdaP*, Δ*tdaQ*, and Δ*tdaQ*Δ*tdaI*, we successfully identified five novel ETP derivatives, designated as **4**–**7** and **4′**. Compounds **4** and **4′** carrying α-hydroxyl, α′-thiomethyl, and β′-acetoxy groups are a pair of isomers with different configurations at β′ position. The structures of **4** and **4′** are similar with the previously reported pseuboydone C, which harbors α-hydroxyl and α′-thiomethyl groups and displays significant cytotoxicity against the Sf9 cells from the fall armyworm *Spodoptera frugiperda* [36,37]. Compound **5** possesses the typical α, β′-disulfide fused with the spirofuran ring and dihydroxy substitutions at C3 and C7′. Compounds **6** and **7** are cFF derivatives featuring a C2-thiomethyl group, a C3′-thiocarbonyl or carbonyl group, and a spirofuran ring system. Based on detailed structural elucidation and biosynthetic pathway of **1**–**3** in *T. hypoxylon*, we also proposed the formation of **4**–**7** and **4′**. Our findings significantly expand the known structural diversity of ETPs, particularly emphasizing new modifications at the C3 and C3′ positions of the cFF core. Furthermore, our results prove again the remarkable catalytic promiscuity of Tda enzymes and enhance our understanding of ETP biosynthesis in fungi. Combined with the previous studies, a vast number of ETPs and their derivatives have been identified in the agriculturally beneficial fungi *Trichoderma* spp. [9,20,26,38,39]. We speculate that such diverse compounds could be important for the antagonism of *Trichoderma* spp. towards other species. Further studies could focus on the specific roles of diverse ETPs on *Trichoderma* spp. and provide new insights for bioactive SMs in biocontrol applications.

## Figures and Tables

**Table 1:** NMR data of **4**, **4′**, and **5** in DMSO-*d*_6_ (500 MHz for ^1^H NMR and 125 MHz for ^13^C NMR).

No.	4		4′		No.	5	
	δ_C_, Type	δ_H_ (*J*, Hz)	δ_C_, Type	δ_H_ (*J*, Hz)		δ_C_, Type	δ_H_ (*J*, Hz)
1	167.1, C	-	167.0, C	-	1	167.3, C	-
2	82.6, C	-	81.5, C	-	2	74.6, C	-
3	44.6, CH_2_	3.45 (d, *J* = 12.6) 2.82 (d, *J* = 12.6)	44.1, CH_2_	3.45 (d, *J* = 12.6) 2.83 (d, *J* = 12.6)	3	71.6, CH	5.11 (s)
4	135.4, C	-	135.1, C	-	4	139.6, C	-
5	130.8, CH	7.13–7.35 (m ^a^)	130.4, CH	7.13–7.35 (m ^a^)	5	128.9, CH	7.58–7.62 (m)
6	128.3, CH	7.13–7.35 (m ^a^)	128.3, CH	7.13–7.35 (m ^a^)	6	127.3, CH	7.24–7.34 (m ^b^)
7	128.7, CH	7.13–7.35 (m ^a^)	128.7, CH	7.13–7.35 (m ^a^)	7	127.6, CH	7.24–7.34 (m ^b^)
8	128.3, CH	7.13–7.35 (m ^a^)	128.3, CH	7.13–7.35 (m ^a^)	8	127.3, CH	7.24–7.34 (m ^b^)
9	130.8, CH	7.13–7.35 (m ^a^)	130.4, CH	7.13–7.35 (m ^a^)	9	128.9, CH	7.58–7.62 (m)
1′	165.3, C	-	164.6, C	-	1′	165.4, C	-
2′	71.2, C	-	58.9, C	-	2′	96.3, C	-
3′	77.2, CH	5.82 (s)	76.6, CH	5.81 (s)	3′	54.4, CH	4.60 (d, *J* = 1.2)
4′	134.1, C	-	134.0, C	-	4′	113.7, C	-
5′	128.0, CH	7.14–7.36 (m ^a^)	128.3, CH	7.13–7.35 (m ^a^)	5′	146.3, C	-
6′	127.9, CH	7.14–7.36 (m ^a^)	127.9, CH	7.13–7.35 (m ^a^)	6′	129.7, C	-
7′	126.7, CH	7.13–7.35 (m ^a^)	126.7, CH	7.13–7.35 (m ^a^)	7′	148.3, C	-
8′	127.9, CH	7.13–7.35 (m ^a^)	127.9, CH	7.13–7.35 (m ^a^)	8′	109.0, CH	6.38 (d, *J* = 8.0)
9′	128.0, CH	7.13–7.35 (m ^a^)	128.3, CH	7.13–7.35 (m ^a^)	9′	113.0, CH	6.43 (dd, *J* = 8.0, 1.2)
11′	168.7, C	-	168.7, C	-	10-NH	-	9.48 (s)
12′	20.5, CH_3_	2.03 (s)	20.3, CH_3_	1.56 (s)	10′-NH	-	9.34 (s)
10-NH	-	9.14 (s)	-	8.95 (s)	3-OH	-	6.19 (s)
10′-NH	-	7.81 (s)	-	7.56 (s)	6′-OH	-	n.d.
2-OH	-	6.74 (s)	-	6.86 (s)	7′-OH	-	n.d.
2′-SCH_3_	9.6, CH_3_	0.86 (s)	12.3, CH_3_	2.08 (s)			

[a, b] signal overlapped; [n.d.] not detected.

**Table 2:** NMR data of **6** and **7** in DMSO-*d*_6_ (500 MHz for ^1^H NMR and 125 MHz for ^13^C NMR).

No.	6		7	
	δ_C_, Type	δ_H_ (*J*, Hz)	δ_C_, Type	δ_H_ (*J*, Hz)
1	165.8, C	-	165.5, C	-
2	67.6, C	-	67.7, C	-
3	43.9, CH_2_	3.04 (d, *J* = 13.0) 3.50 (d, *J* = 13.0)	43.4, CH_2_	3.03 (d, *J* = 13.3) 3.50 (d, *J* = 13.3)
4	134.3, C	-	134.5, C	-
5	130.4, CH	7.23–7.25 (m ^a^)	130.4, CH	7.23–7.25 (m ^a^)
6	128.3, CH	7.26–7.34 (m ^b^)	128.3, CH	7.26–7.35 (m ^b^)
7	127.2, CH	7.26–7.34 (m ^b^)	127.3, CH	7.26–7.35 (m ^b^)
8	128.3, CH	7.26–7.34 (m ^b^)	128.3, CH	7.26–7.35 (m ^b^)
9	130.4, CH	7.23–7.25 (m ^a^)	130.4, CH	7.23–7.25 (m ^a^)
1′	160.2, C	-	160.4, C	-
2′	99.0, C	-	88.3, C	-
3′	227.5, C	-	194.9, C	-
4′	134.0, C	-	119.9, C	-
5′	160.1, C	-	160.5, C	-
6′	143.6, C	-	143.5, C	-
7′	124.7, CH	7.18 (dd, *J* = 7.8, 1.2)	124.4, CH	7.15 (dd, *J* = 7.7, 1.2)
8′	123.7, CH	6.92 (t, *J* = 7.8)	123.3, CH	6.96 (t, *J* = 7.7)
9′	114.0, CH	7.08 (dd, *J* = 7.8, 1.2)	114.1, CH	7.03 (dd, *J* = 7.7, 1.2)
10-NH	-	9.54 (s)	-	9.67 (s)
10′-NH	-	9.49 (s)	-	9.41 (s)
2-SCH_3_	13.5, CH_3_	2.30 (s)	13.0, CH_3_	2.31 (s)
6′-OH	-	10.12 (s)	-	10.15 (s)

[a, b] signal overlapped.

## Data Availability

The original contributions presented in this study are included in the article/Supplementary Materials. Further inquiries can be directed to the corresponding authors.

## Supplementary Materials

The following are available online at https://www.mdpi.com/article/10.3390/jof11040241/s1, Table S1: Strains used in this study; Table S2: HR-ESI-MS data of the compounds **4**–**7** and **4′**; Figure S1: *Tda* gene cluster from *Trichoderma hypoxylon*; Figure S2: ETP derivatives previously isolated from *T. hypoxylon* strains; and Figures S3–S6: NMR spectra of **4**–**7** and **4′** in DMSO-*d_6_*. References [16,27,40] are cited in the supplementary materials.
