# Supplementary material for: Novel Epidithiodiketopiperazine Derivatives in the Mutants of the Filamentous Fungus Trichoderma hypoxylon
Source: J Fungi (Basel). 2025 Mar 22;11(4):241. doi: 10.3390/jof11040241 (PMC12028603; doi:10.3390/jof11040241)
Supplement: Supplementary file 1 [file jof-11-00241-s001.zip › jof-3505536-supplementary.pdf]

Supplementary Materials for

Novel Epidithiodiketopiperazine Derivatives in the Mutants of the Filamentous Fungus *Trichoderma hypoxylon*

Zedong Ren [1,2,†], Yuanyuan Li [2,3,†], Peng-Lin Wei [2,3], Shengquan Zhang [2,4], Dong Wang [1,5], Jie Fan [2,*] and Wen-Bing Yin [2,3,*]

[1] School of Traditional Chinese Materia Medica, Shenyang Pharmaceutical University, Shenyang 110016, China; ren102323@163.com (Z.R.); dongwang@syphu.edu.cn (D.W.)
[2] State Key Laboratory of Mycology, Institute of Microbiology, Chinese Academy of Sciences, Beijing 100101, China; 15008151347@163.com (Y.L.); wpl0816@126.com (P.-L.W.); 13140607588@163.com (S.Z.)
[3] Medical School, University of Chinese Academy of Sciences, Beijing 100049, China
[4] Shandong Academy of Medical Sciences, Shandong First Medical University, Jinan 250117, China
[5] Laboratory of China-Korea Molecular Pharmacognosy, Shenyang Pharmaceutical University, Shenyang 110016, China
***** Correspondence: fanjie@im.ac.cn (J.F.); yinwb@im.ac.cn (W.-B.Y.); Tel.: +86-010-64806170 (W.-B.Y.)
[†] These authors contributed equally to this work.

Table of Contents

Supplementary tables 3

Table S1. Strains used in this study. 3

Table S2. HR-ESI-MS data of compounds **4**–**7** and **4'**. 4

Supplementary figures 5

Figure S1. *Tda* gene cluster from *Trichoderma hypoxylon* 5

Figure S2. ETP derivatives previously isolated from *T. hypoxylon* strains. 6

Figure S3 (A). ^{1}H NMR spectrum of **4** and **4'** in DMSO-d_6 (500 MHz). 8

Figure S3 (B). ^{13}C NMR spectrum of **4** and **4'** in DMSO-d_6 (125 MHz). 8

Figure S3 (C). HSQC spectrum of **4** and **4'** in DMSO-d_6. 9

Figure S3 (D). HMBC spectrum of **4** and **4'** in DMSO-d_6. 9

Figure S3 (E). ^{1}H-^{1}H COSY spectrum of **4** and **4'** in DMSO-d_6. 10

Figure S4 (A). ^{1}H NMR spectrum of **5** in DMSO-d_6 (500 MHz). 10

Figure S4 (B). ^{13}C NMR spectrum of **5** in DMSO-d_6 (125 MHz). 11

Figure S4 (C). HSQC spectrum of **5** in DMSO-d_6. 11

Figure S4 (D). HMBC spectrum of **5** in DMSO-d_6. 12

Figure S4 (E). ^{1}H-^{1}H COSY spectrum of **5** in DMSO-d_6. 12

Figure S5 (A). ^{1}H NMR spectrum of **6** in DMSO-d_6 (500 MHz). 13

Figure S5 (B). ^{13}C NMR spectrum of **6** in DMSO-d_6 (125 MHz). 13

Figure S5 (C). HSQC spectrum of **6** in DMSO-d_6. 14

Figure S5 (D). HMBC spectrum of **6** in DMSO-d_6. 14

Figure S5 (E). ^{1}H-^{1}H COSY spectrum of **6** in DMSO-d_6. 15

Figure S6 (A). ^{1}H NMR spectrum of **7** in DMSO-d_6 (500 MHz). 15

Figure S6 (B). ^{13}C NMR spectrum of **7** in DMSO-d_6 (125 MHz). 16

Figure S6 (C). HSQC spectrum of **7** in DMSO-d_6. 16

Figure S6 (D). HMBC spectrum of **7** in DMSO-d_6. 17

Figure S6 (E). ^{1}H-^{1}H COSY spectrum of **7** in DMSO-d_6. 17

References 18

Supplementary tables

Table S1. Strains used in this study.

Strains	Genotype	Source
TYHL26	Δ*tri5::neo*	[1]
TYHL41	Δ*tdaP::hph*, Δ*tri5::neo*	[2]
TYPL43	Δ*tdaQ::hph*, Δ*tri5::neo*	[3]
TYYH3	Δ*tri5::neo*, Δ*thlig4*, Δ*pyr4*	[2]
TYPL52	Δ*tdaQ::hph*, Δ*tdaI::AfpyrG*, Δ*tri5::neo*, Δ*thlig4*, Δ*pyr4*	[2]

Table S2. HR-ESI-MS data of compounds **4–7** and **4'**.

No.	Compound	Isolated from	Physical description	Chemical formula	HR-ESI-MS of $[M + Na]^+$		Deviation (ppm)
					Calculated	Measured	
1	**4**	Δ*tdaP*Δ*tri5*	White powder	$C_{21}H_{22}N_2O_5S$	437.1142	437.1149	-1.60
2	**4'**	Δ*tdaP*Δ*tri5*	White powder	$C_{21}H_{22}N_2O_5S$	437.1142	437.1133	2.06
3	**5**	Δ*tdaQ*Δ*tri5*	White powder	$C_{18}H_{14}N_2O_6S_2$	441.0185	441.0188	-0.68
4	**6**	Δ*tdaQ*Δ*tdaI* Δ*tri5*Δ*thlig4*	Yellow powder	$C_{19}H_{16}N_2O_4S_2$	423.0444	423.0453	-2.13
5	**7**	Δ*tdaQ*Δ*tdaI* Δ*tri5*Δ*thlig4*	Yellow powder	$C_{19}H_{16}N_2O_5S$	407.0672	407.0674	-0.49

Supplementary figures

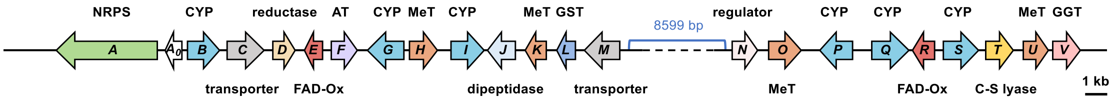

Figure S1. *Tda* gene cluster from *Trichoderma hypoxylon*.NRPS, nonribosomal peptide synthetase; CYP, cytochrome P450 monooxygenase; FAD-Ox, FAD-dependent oxidase; AT, acyltransferase; MeT, methyltransferase; GST, glutathione-*S*-transferase; GGT, glutamyltransferase.

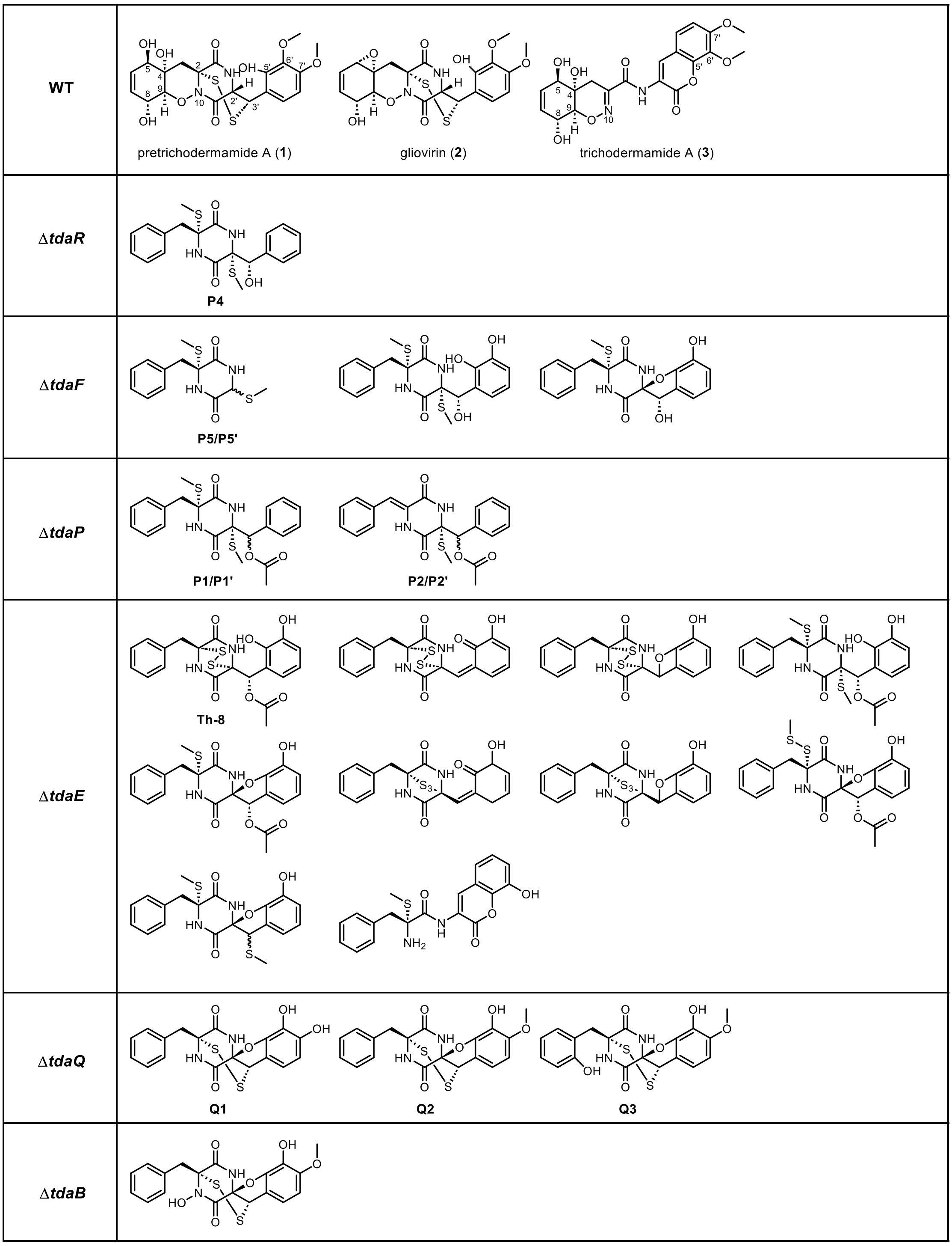

Figure S2. ETP derivatives previously isolated from *T. hypoxylon* strains.

Δ*tdaI*	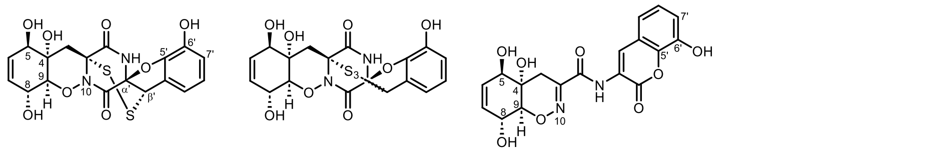
Δ*tdaH*	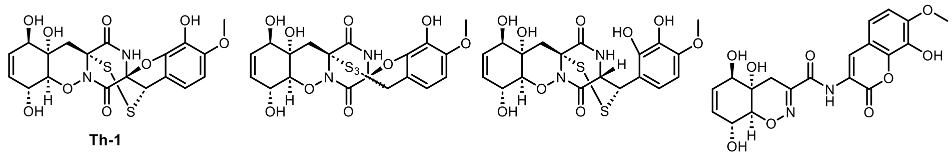 Th-1
Δ*tdaD*	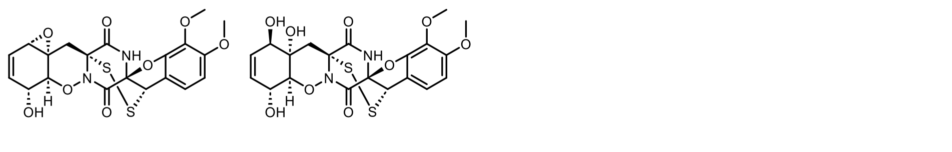
Δ*tdaG*	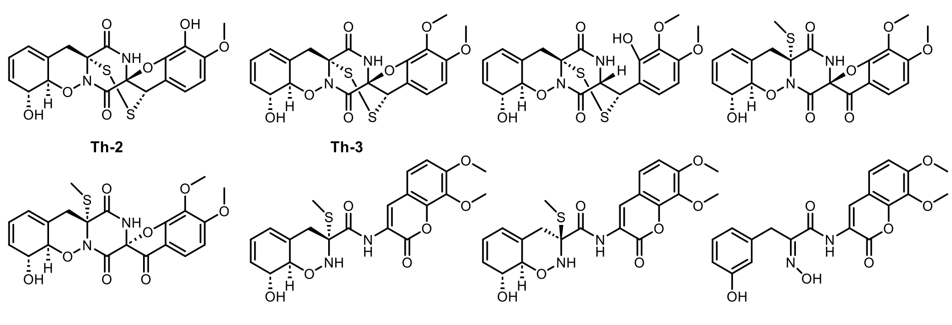 Th-2, Th-3
Δ*tdaI* Δ*tdaQ*	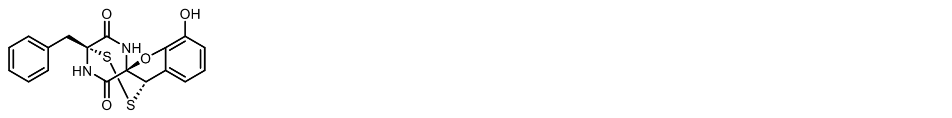 QI1
Δ*tdaI* Δ*tdaG*	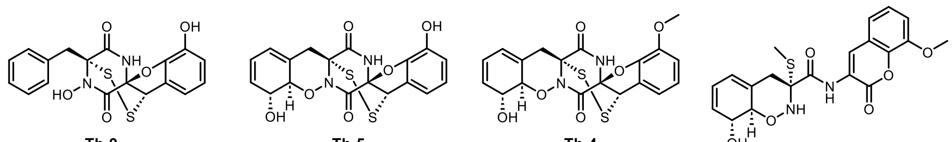 Th-9, Th-5, Th-4
Δ*tdaH* Δ*tdaG*	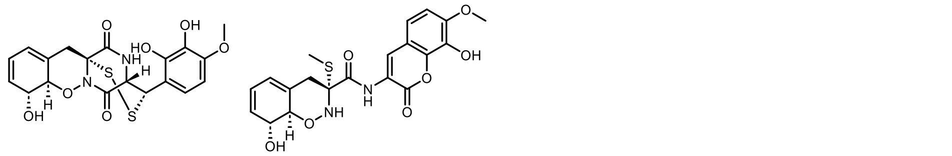

Figure S2. ETP derivatives previously isolated from *T. hypoxylon* strains (Continued).

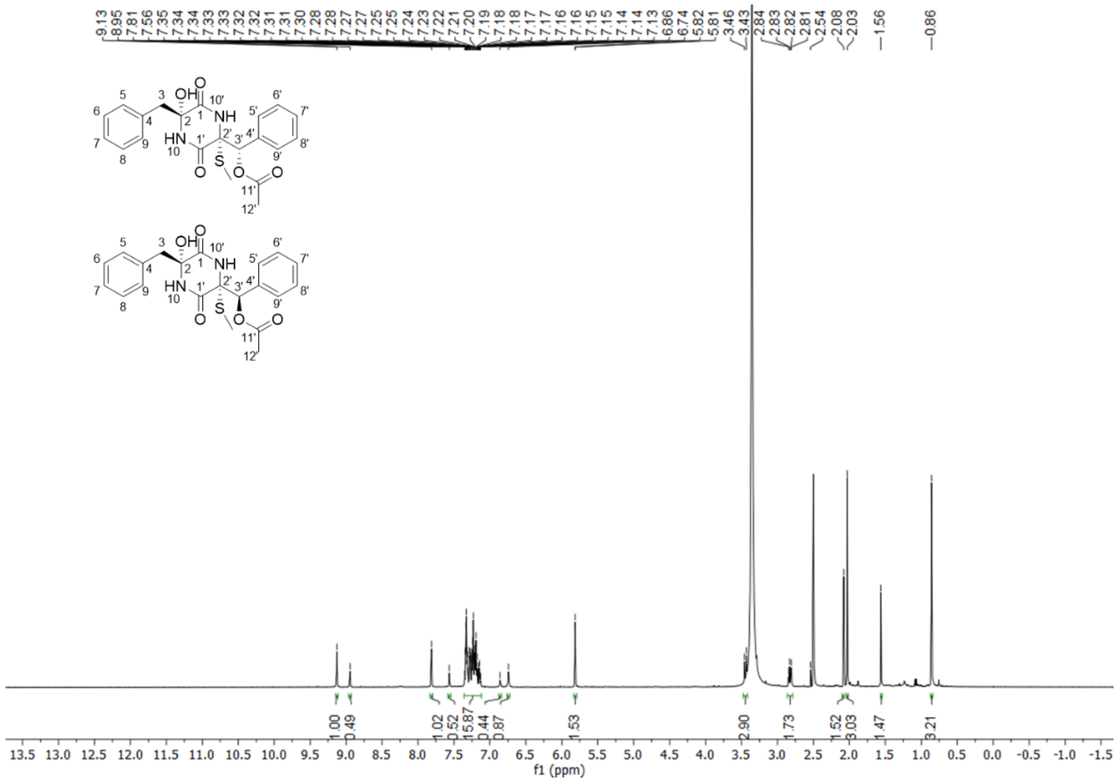

Figure S3 (A). ^{1}H NMR spectrum of **4** and **4'** in DMSO-d_6 (500 MHz).

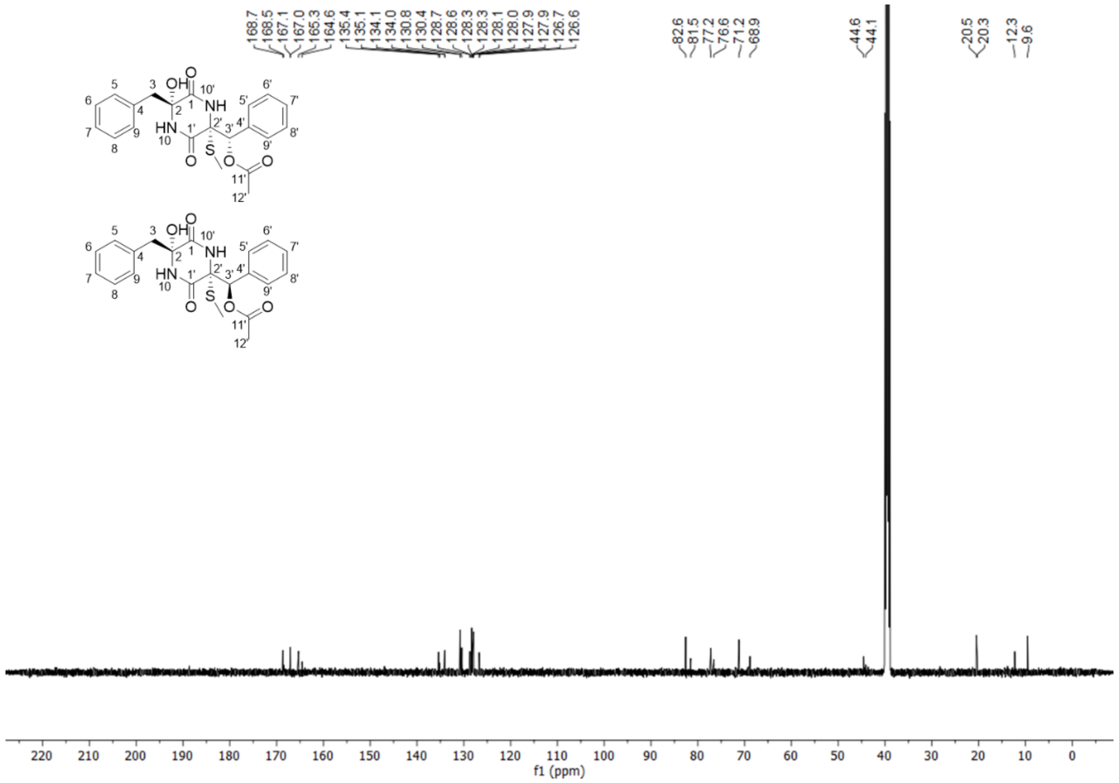

Figure S3 (B). ^{13}C NMR spectrum of **4** and **4'** in DMSO-d_6 (125 MHz).

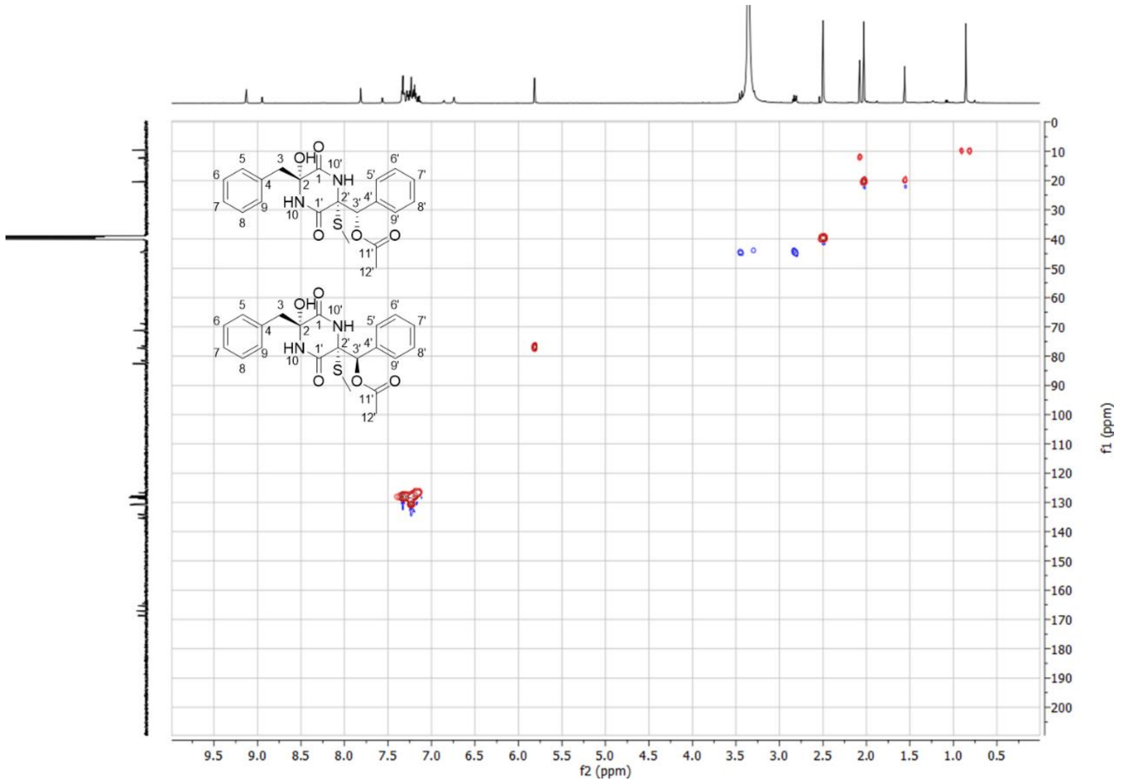

Figure S3 (C). HSQC spectrum of **4** and **4'** in DMSO-d_6.

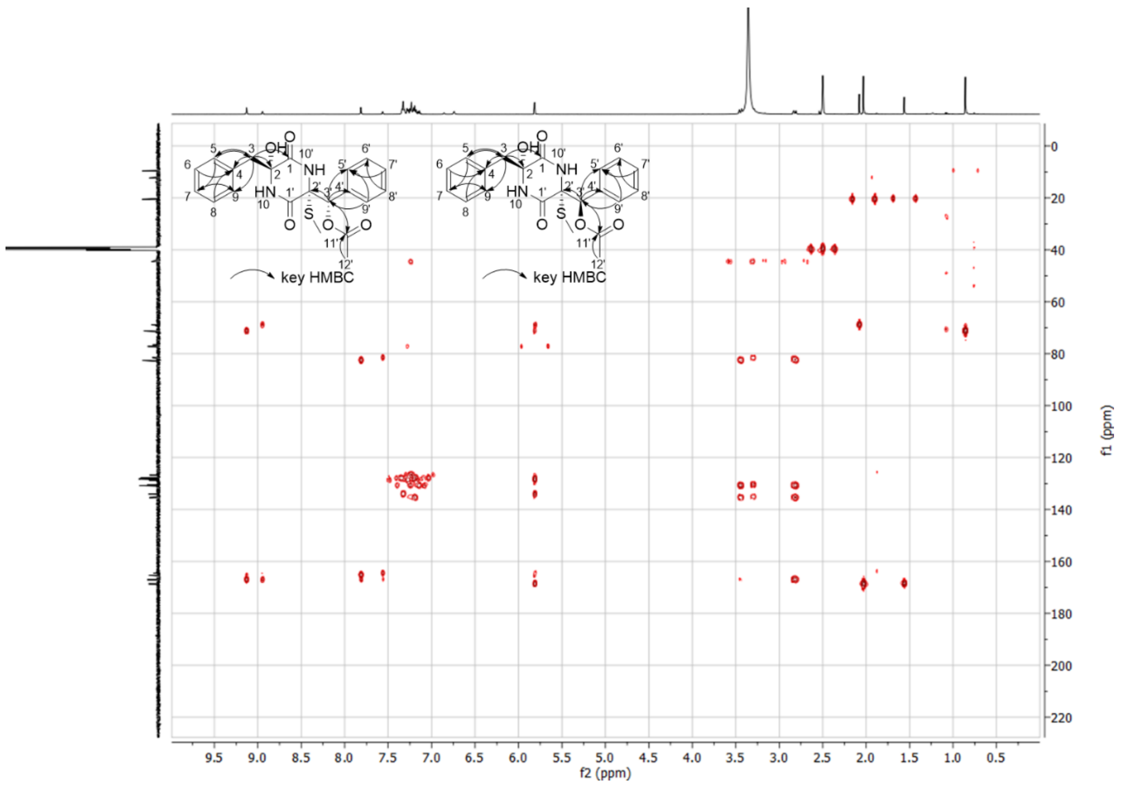

Figure S3 (D). HMBC spectrum of **4** and **4'** in DMSO-d_6.

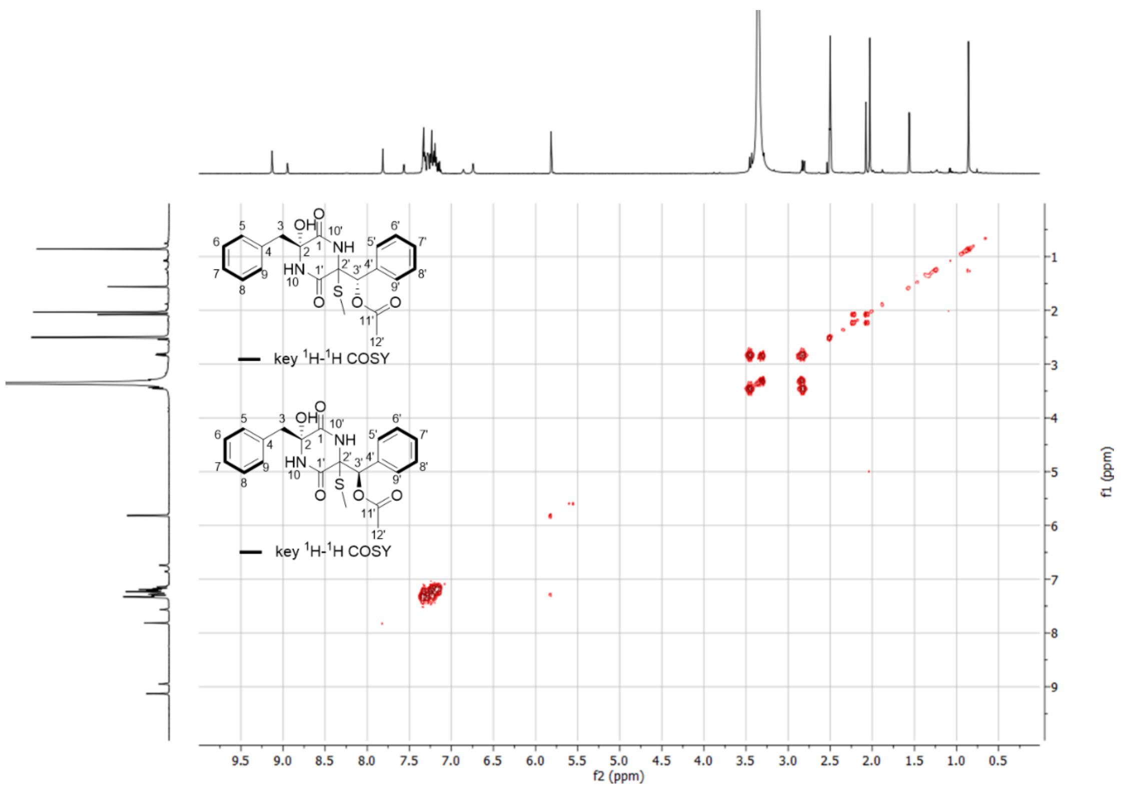

Figure S3 (E). ^{1}H-^{1}H COSY spectrum of **4** and **4'** in DMSO-d_6.

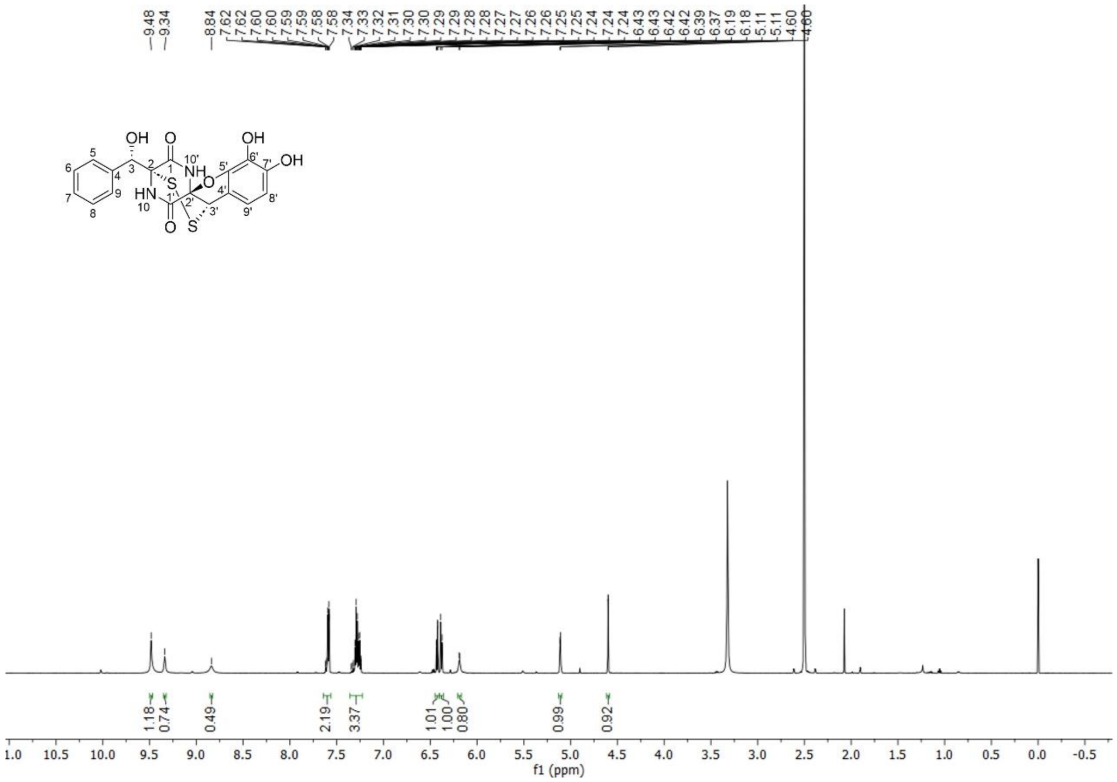

Figure S4 (A). ^{1}H NMR spectrum of **5** in DMSO-d_6 (500 MHz).

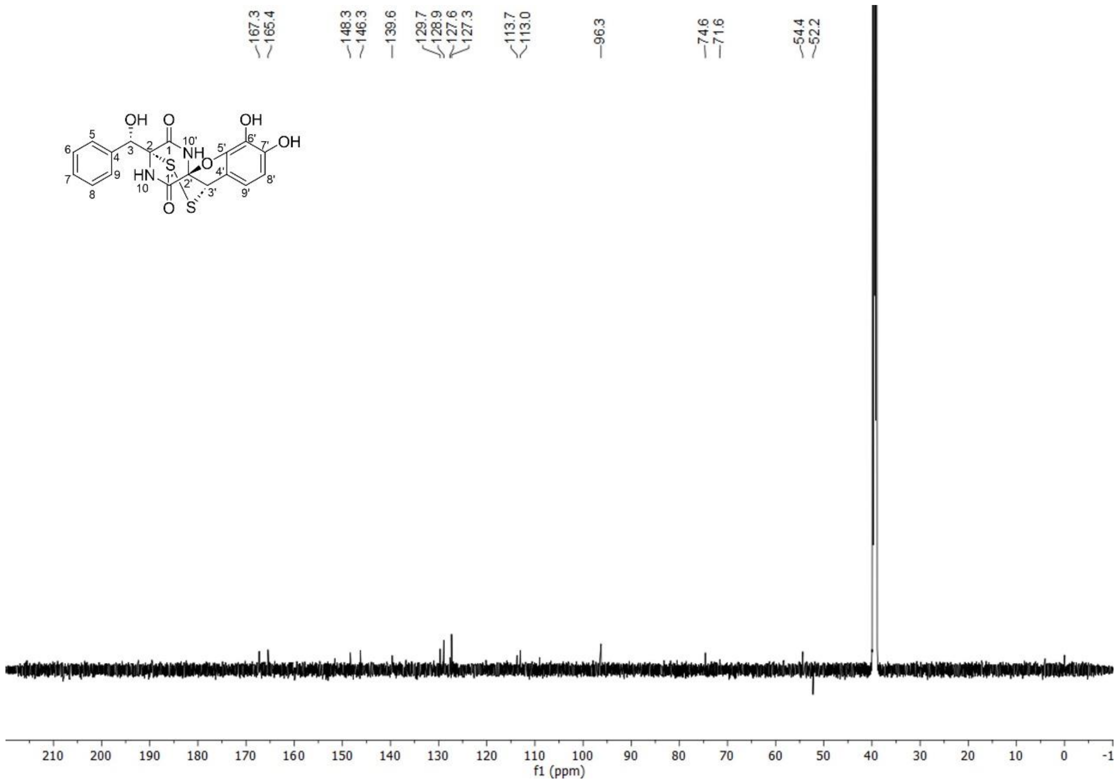

Figure S4 (B). ^{13}C NMR spectrum of **5** in DMSO-d_6 (125 MHz).

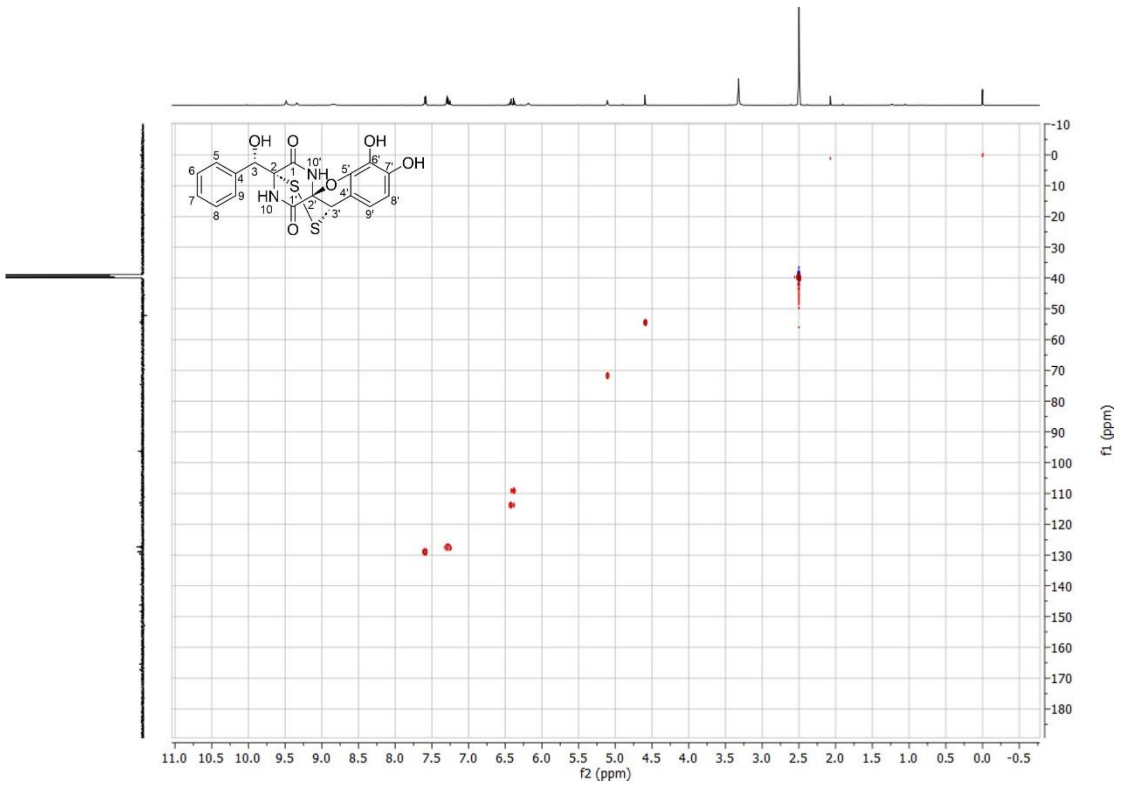

Figure S4 (C). HSQC spectrum of **5** in DMSO-d_6.

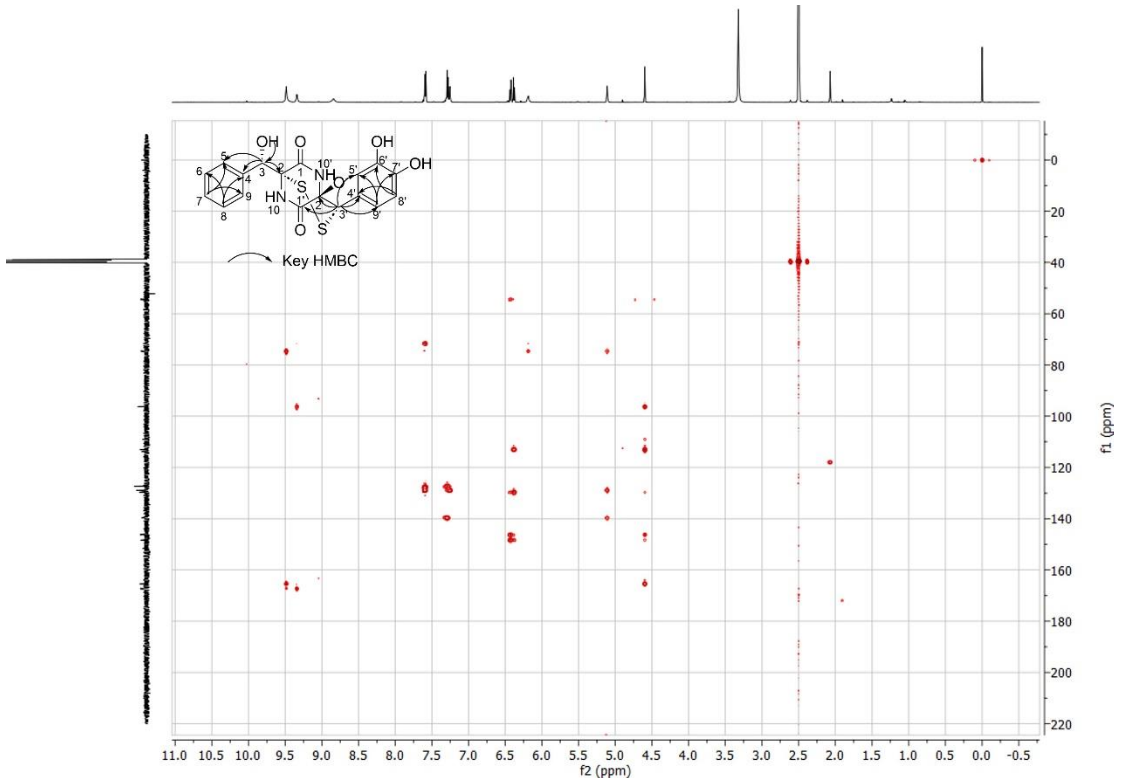

Figure S4 (D). HMBC spectrum of **5** in DMSO-d_6.

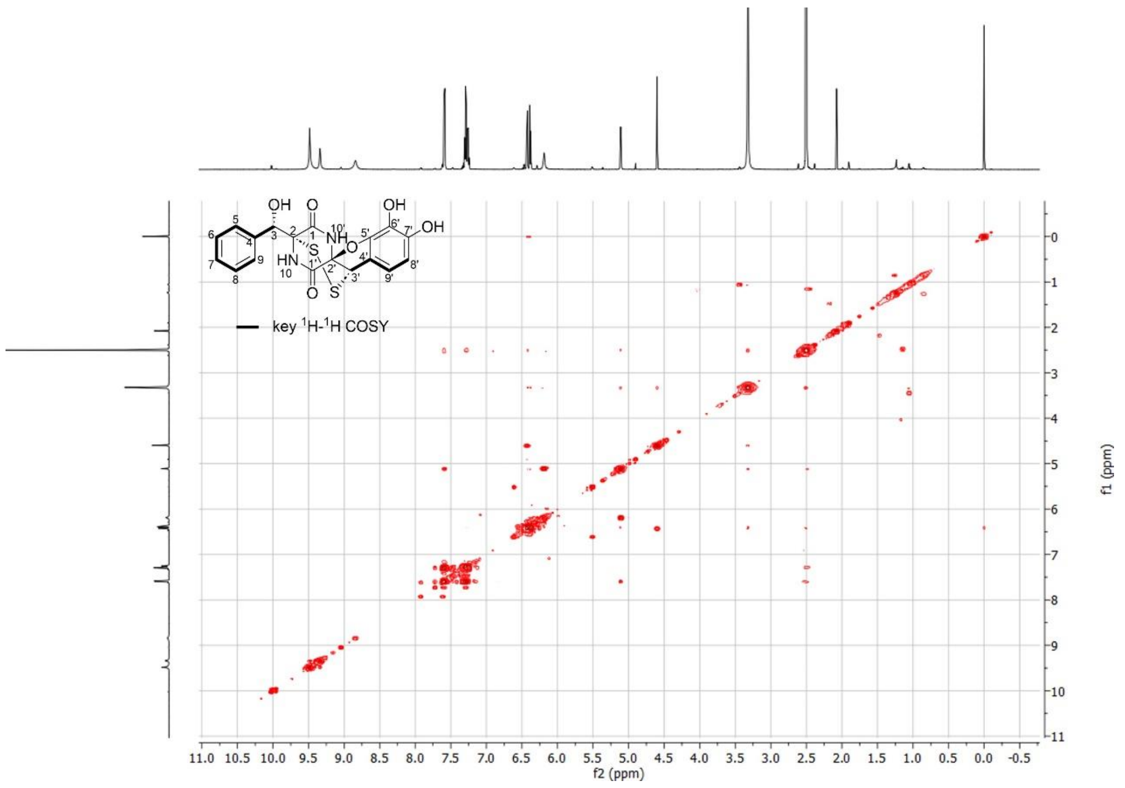

Figure S4 (E). ^{1}H-^{1}H COSY spectrum of **5** in DMSO-d_6.

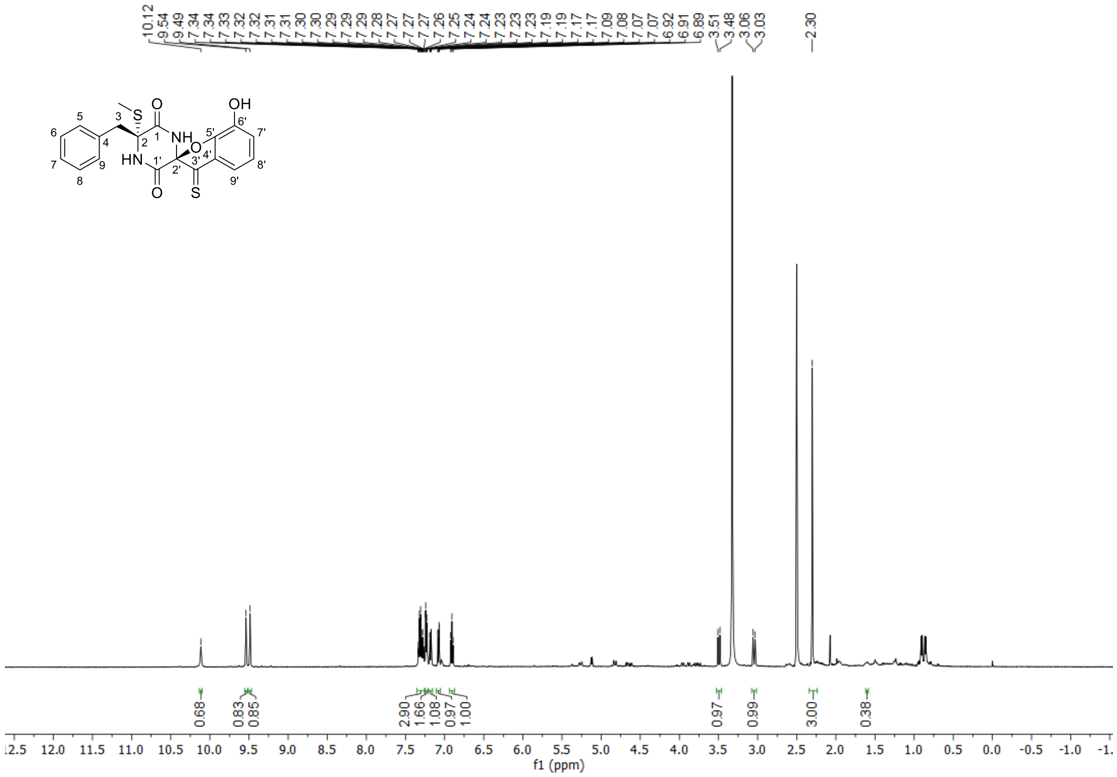

Figure S5 (A). ^{1}H NMR spectrum of **6** in DMSO-d_6 (500 MHz).

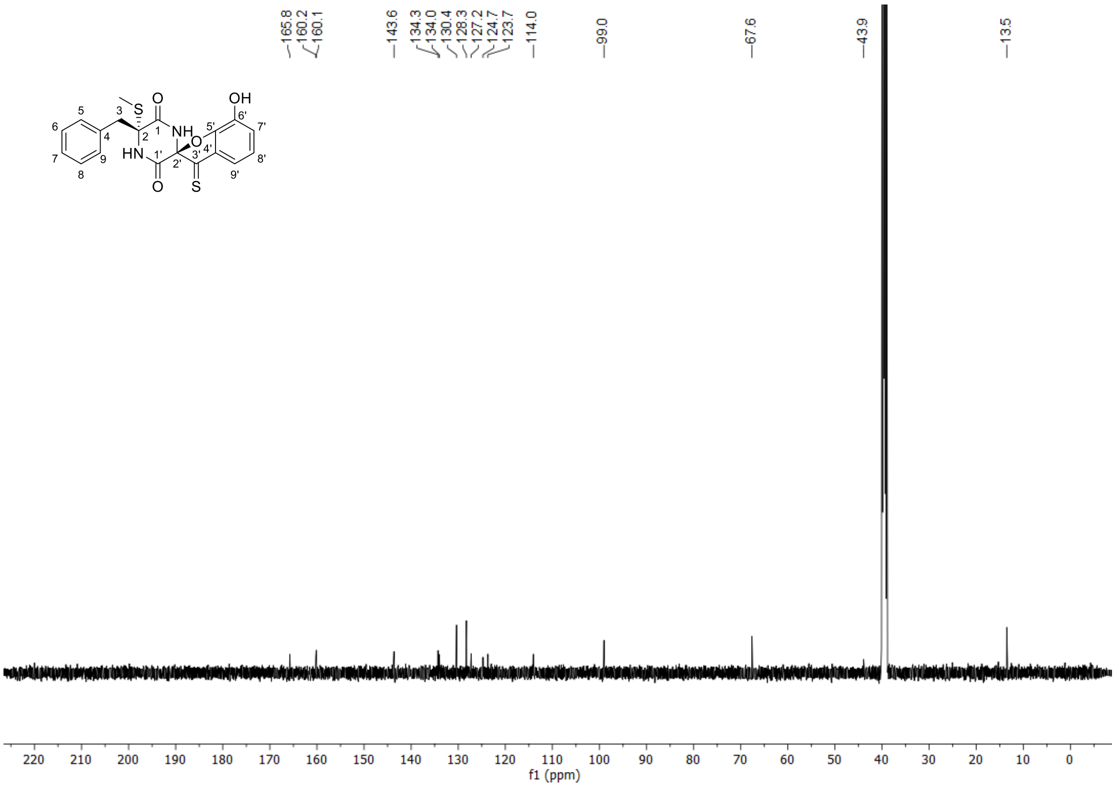

Figure S5 (B). ^{13}C NMR spectrum of **6** in DMSO-d_6 (125 MHz).

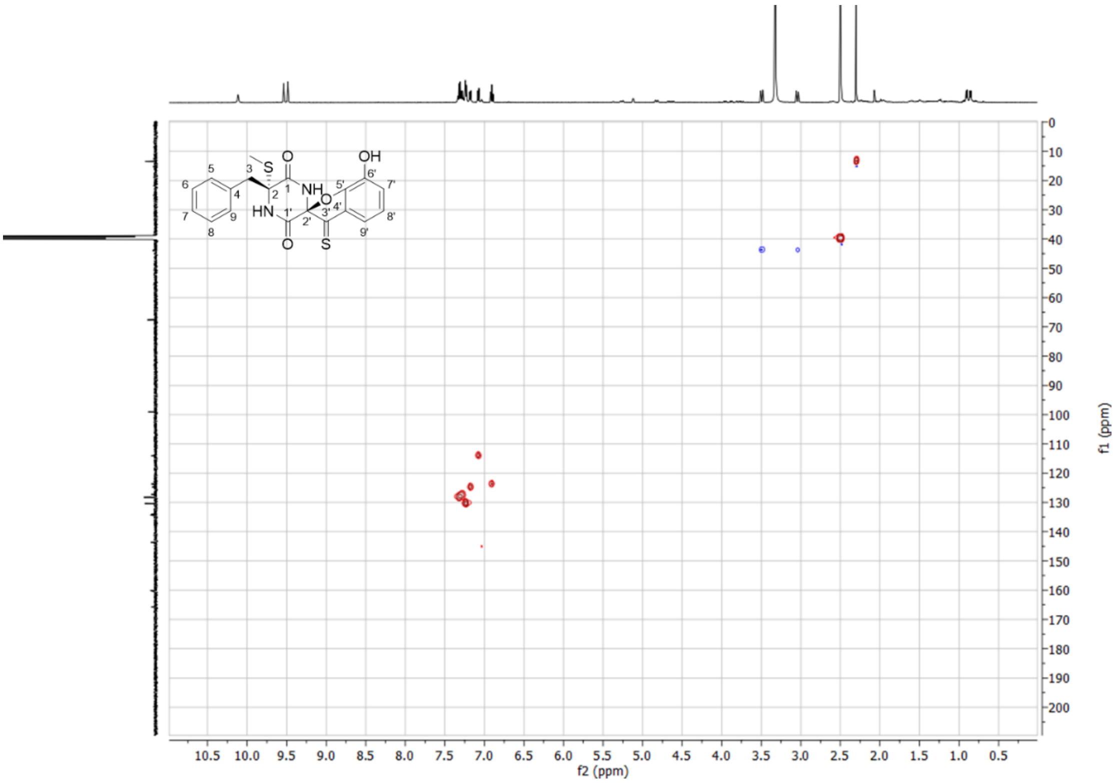

Figure S5 (C). HSQC spectrum of **6** in DMSO-d_6.

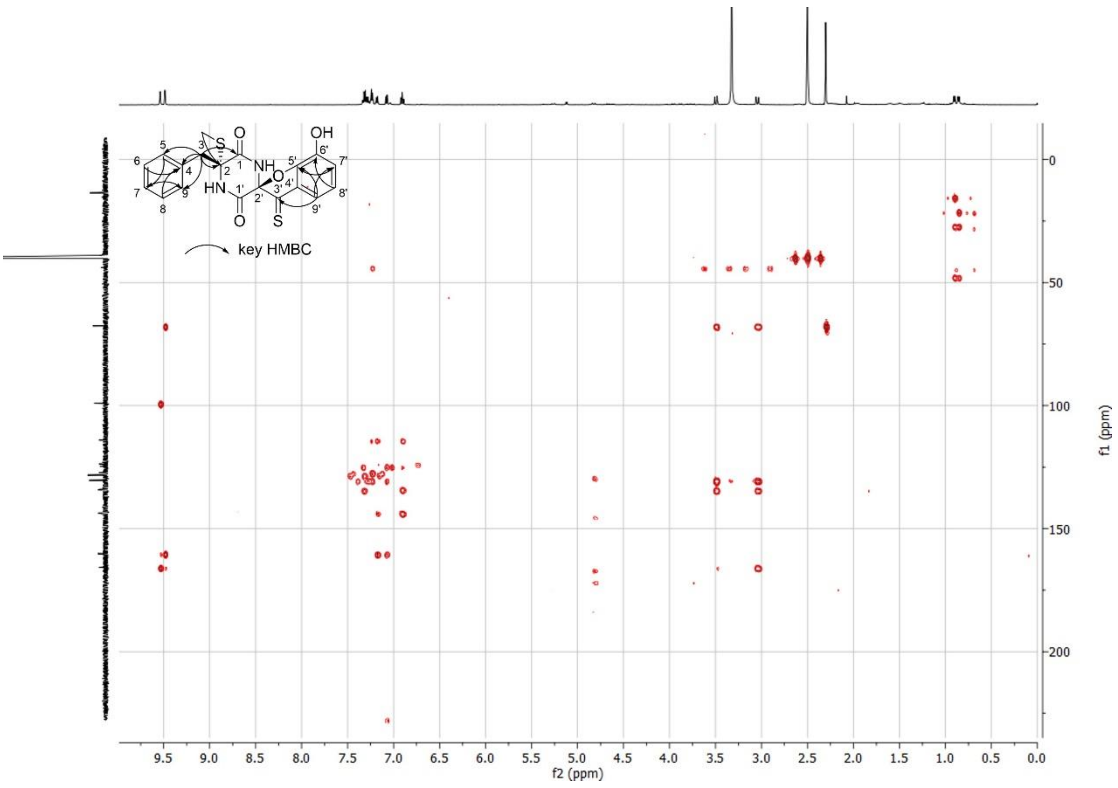

Figure S5 (D). HMBC spectrum of **6** in DMSO-d_6.

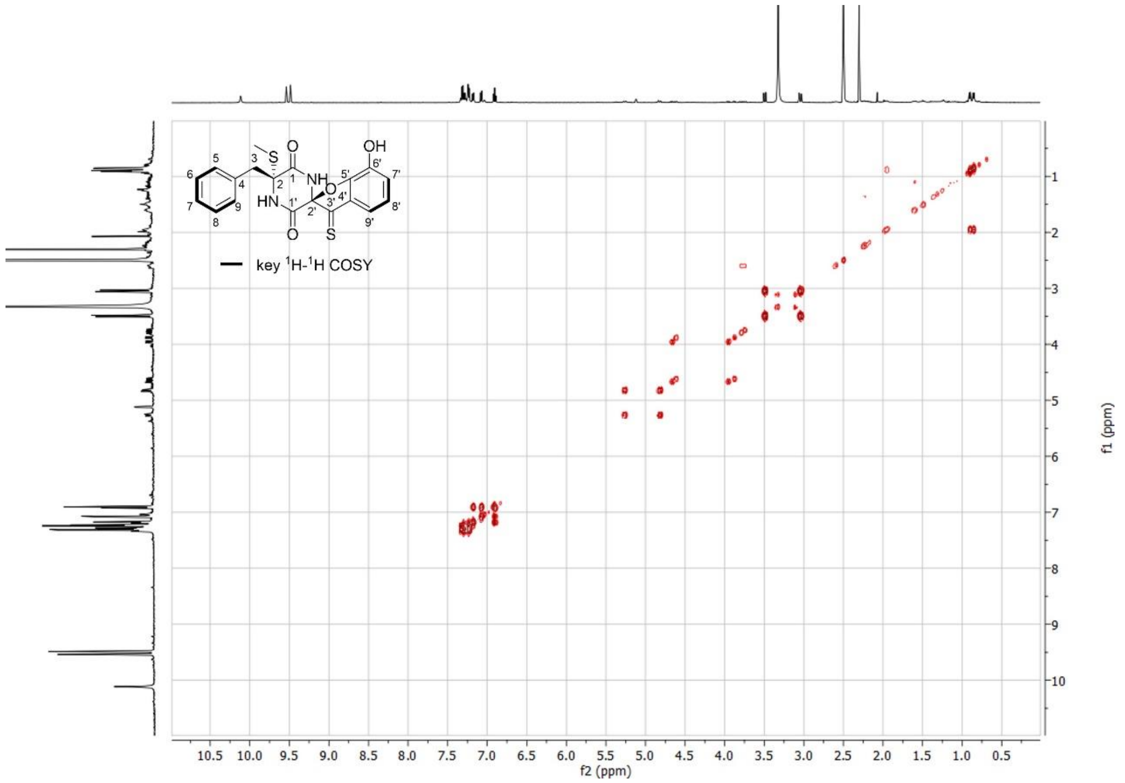

Figure S5 (E). ^{1}H-^{1}H COSY spectrum of **6** in DMSO-d_6.

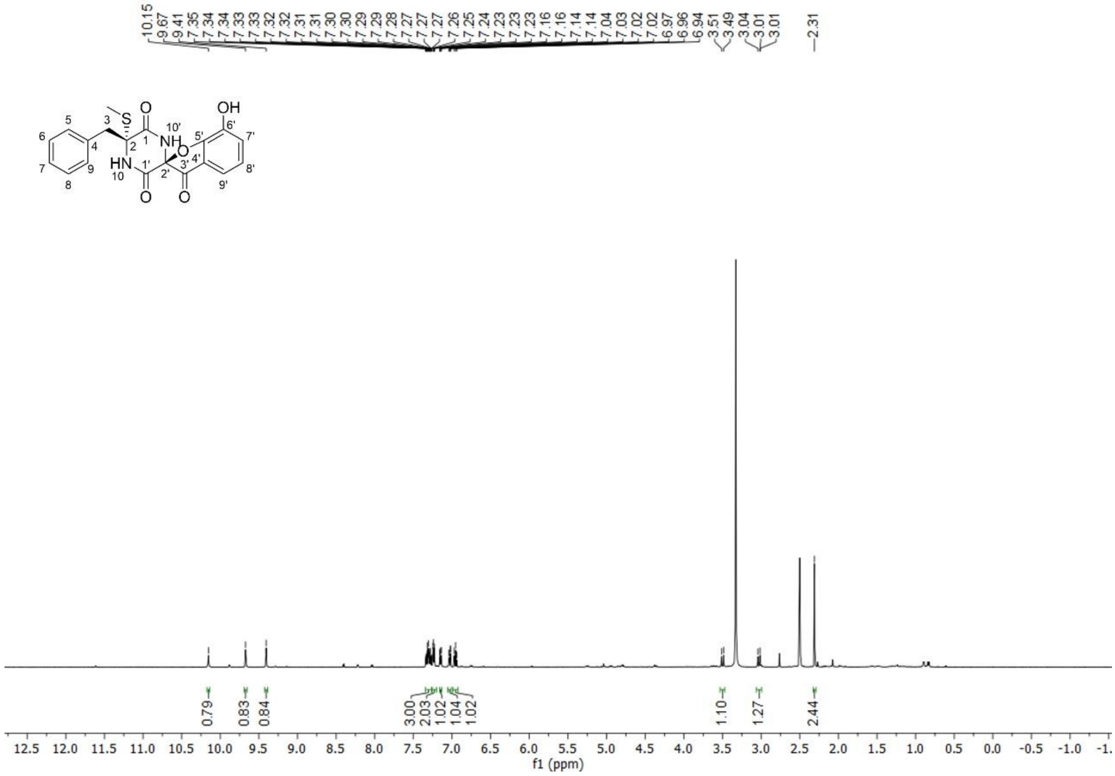

Figure S6 (A). ^{1}H NMR spectrum of **7** in DMSO-d_6 (500 MHz).

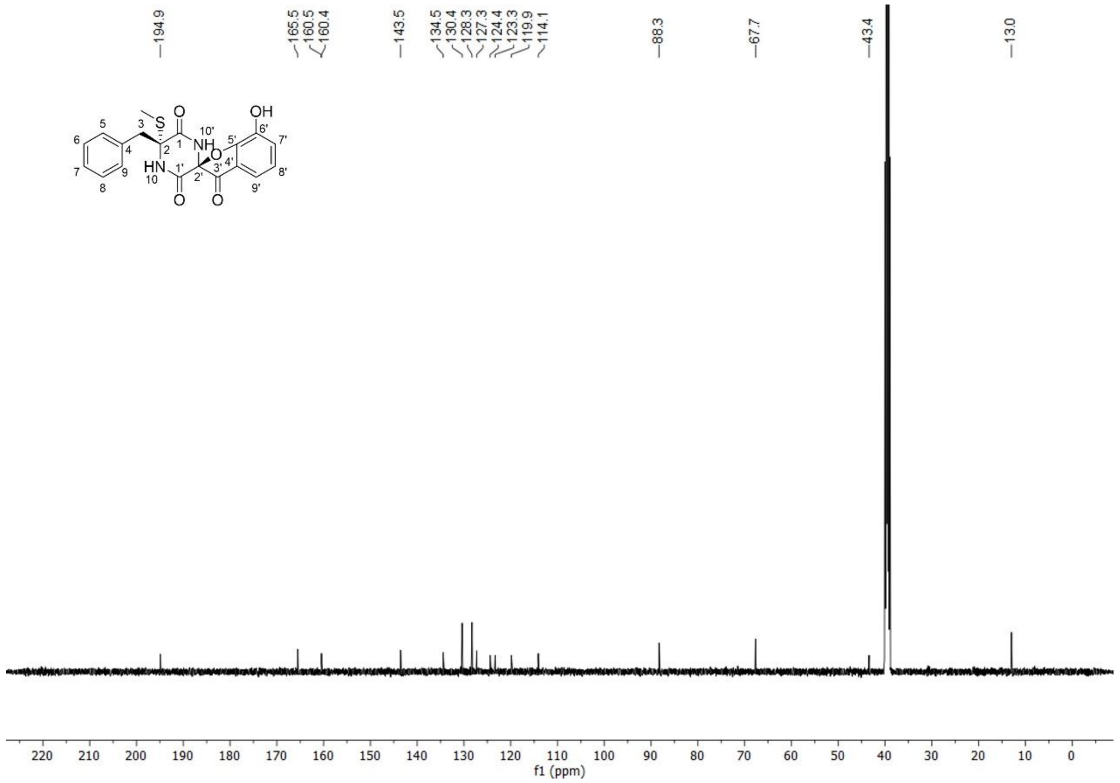

Figure S6 (B). ^{13}C NMR spectrum of **7** in DMSO-d_6 (125 MHz).

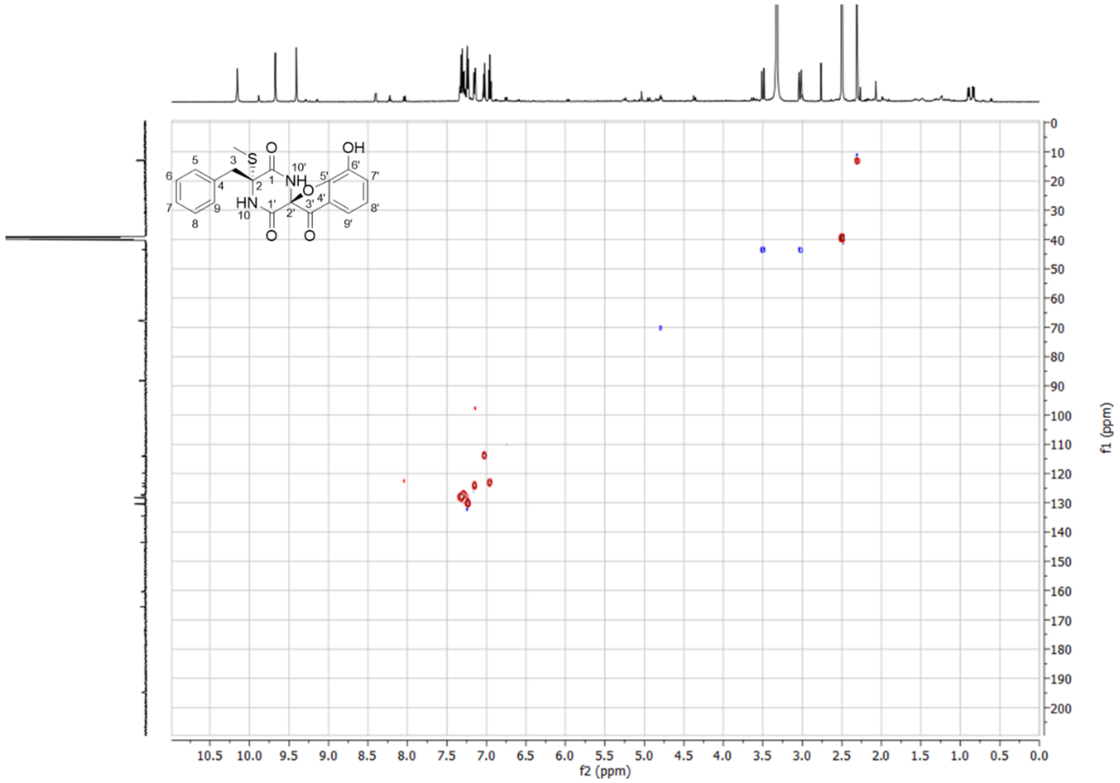

Figure S6 (C). HSQC spectrum of **7** in DMSO-d_6.

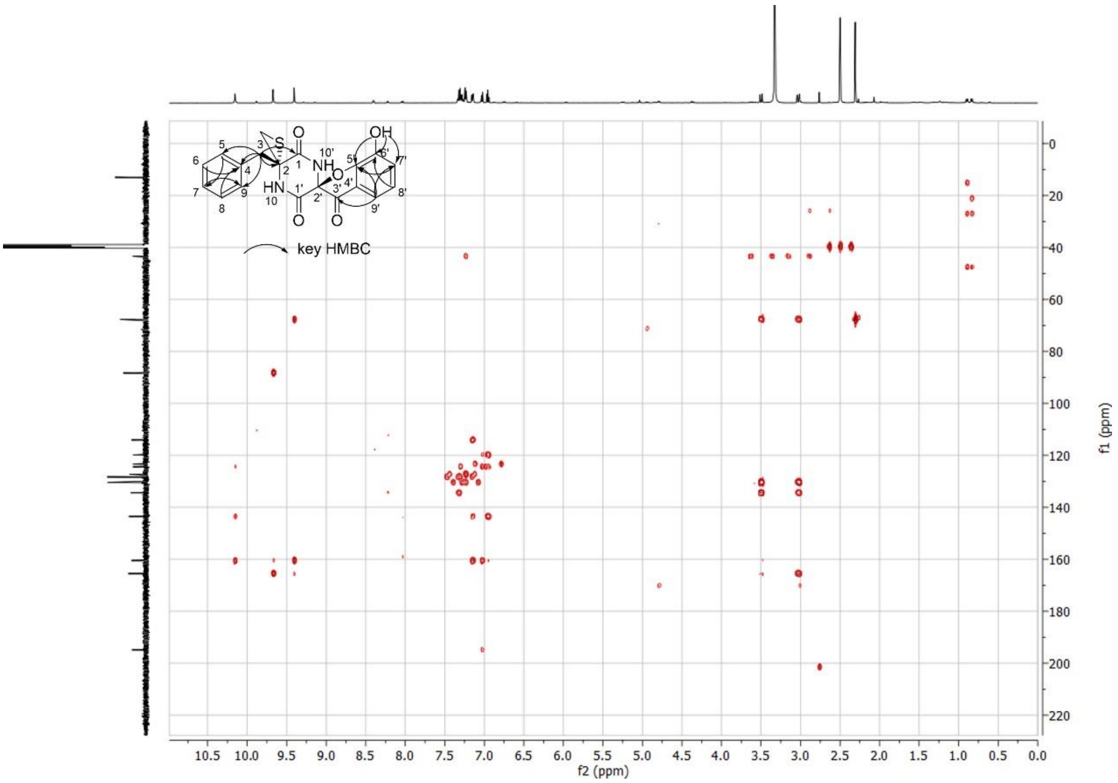

Figure S6 (D). HMBC spectrum of **7** in DMSO-d_6.

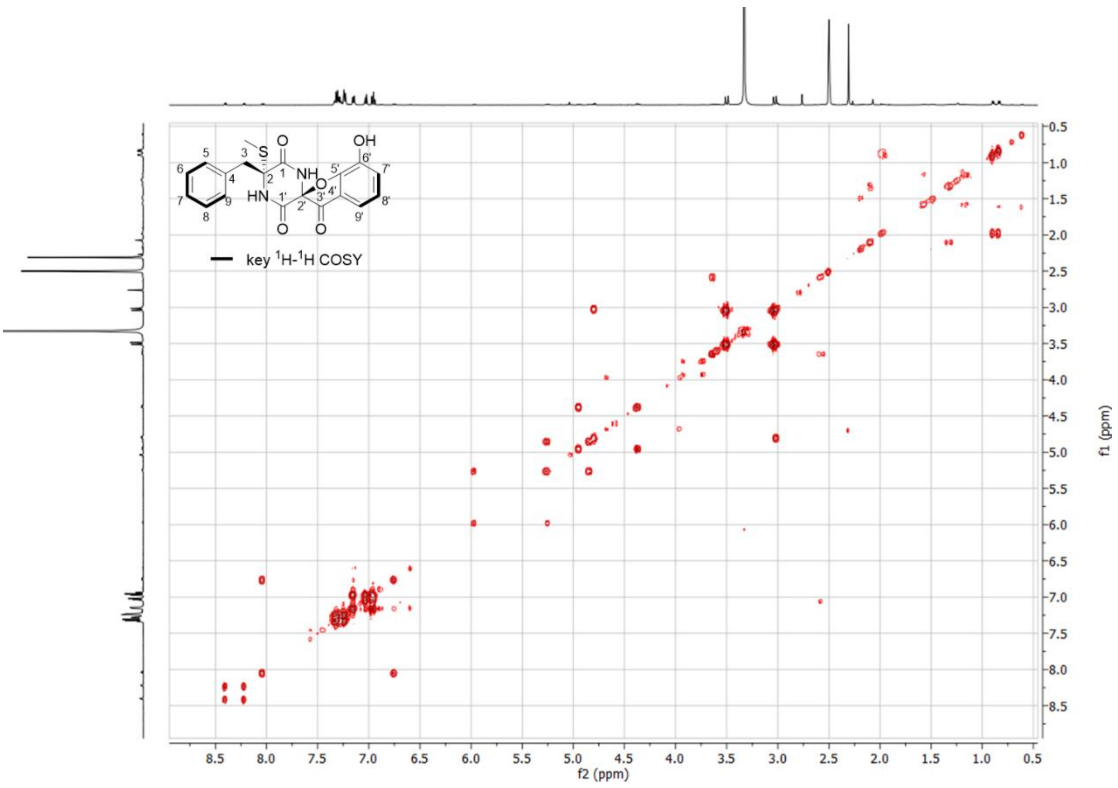

Figure S6 (E). ^{1}H-^{1}H COSY spectrum of **7** in DMSO-d_6.

References

1. Liu, H.; Wang, G.; Li, W.; Liu, X.; Li, E.; Yin, W.-B. A highly efficient genetic system for the identification of a harzianum B biosynthetic gene cluster in *Trichoderma hypoxylon*. *Microbiology* **2018**, *164*, 769-778, doi:10.1099/mic.0.000649.
2. Fan, J.; Ran, H.; Wei, P.-L.; Li, Y.; Liu, H.; Li, S.-M.; Yin, W.-B. An *ortho*-quinone methide mediates disulfide migration in the biosynthesis of epidithiodiketopiperazines. *Angew. Chem. Int. Ed.* **2023**, *62*, e202304252, doi:10.1002/anie.202304252.
3. Fan, J.; Ran, H.; Wei, P.-L.; Li, Y.; Liu, H.; Li, S.-M.; Hu, Y.; Yin, W.-B. Pretrichodermamide A biosynthesis reveals the hidden diversity of epidithiodiketopiperazines. *Angew. Chem. Int. Ed.* **2023**, *62*, e202217212, doi:10.1002/anie.202217212.
